# Single-cell analysis of the progeria arterial wall reveals progerin-induced progressive, cell type-specific dysfunction and somatic mutation accumulation

**DOI:** 10.1186/s13073-026-01719-6

**Published:** 2026-07-31

**Authors:** Lara G. Merino, Gwladys Revêchon, Santhilal Subhash, Fabiana Stefani, Daniel Whisenant, Marianna Skipitari, Quentin Giraud, Lars Muhl, Giuseppe Mocci, Johan Björkegren, Piotr Machtel, Liqun He, Christer Betsholtz, Maria Eriksson

**Affiliations:** 1 Department of Medicine Huddinge, 141 83 Huddinge, Karolinska Institutet Sweden; 2 Department of Biosciences and Bioengineering, Indian Institute of Technology Jammu, Jammu, India; 3 Department of Clinical Medicine, Centre for Cancer Biomarkers (CCBIO), University of Bergen, Bergen, Norway

**Keywords:** Somatic mutations, Cardiovascular disease, Arterial wall, Single-cell RNA sequencing, Smart-seq2, Hutchinson-Gilford progeria syndrome, Premature vascular aging

## Abstract

**Background:**

The premature aging disorder Hutchinson-Gilford Progeria Syndrome (HGPS) is caused by de novo* LMNA* mutations producing the aberrant Lamin A isoform progerin. HGPS patients die from cardiovascular disease, with their arteries showing extensive cellular and structural remodeling, but the mechanisms driving vascular dysfunction are not fully understood.

**Methods:**

To define molecular processes underlying progressive vascular degeneration in HGPS, we performed single-cell RNA-sequencing (scRNA-seq) of aortic arch cells from *Lmna*^*G609G/G609G*^ mice without atheroprone stimuli. These mice carry the murine equivalent of the most common HGPS-causing mutation and faithfully recapitulate the vascular phenotype. Sequencing was performed at multiple ages to capture disease-related and time-dependent transcriptional changes. We used Smart-seq2 for sequencing, due to its high sensitivity and full-length transcript coverage. Histology, immunostaining and in situ hybridization were used for arterial characterization.

**Results:**

The aortic arch of *Lmna*^*G609G/G609G*^ mice exhibited a gradual age-dependent vascular smooth muscle cell (VSMC) loss, accompanied by a transient proliferation surge, and ultimately by increased apoptosis. scRNA-seq identified transcriptionally distinct cell populations with unique features that evolved during disease progression. Disease-enriched VSMCs at early stages were characterized by elevated endoplasmic reticulum (ER) stress. With disease development, these VSMCs further underwent phenotypic switching toward a fibroblast-like state, which was predicted to expand through non-cell-autonomous mechanisms. At later stages, disease-enriched VSMCs upregulated apoptotic gene expression, partially coinciding with sustained ER stress. Furthermore, progeria VSMCs showed an increase in both DNA damage and somatic SNVs, with the increased number of SNVs correlating with high expression of ER stress, ROS and p53-related genes. In contrast, progeria-enriched fibroblasts either became activated or increased their cartilage production and showed a delayed accumulation of somatic SNVs compared to VSMCs, highlighting both a cell-type-specific progerin response and differences in somatic mutation susceptibility.

**Conclusions:**

Our study shows that progerin leads to somatic mutation accumulation particularly in VSMCs, highlighting the need for early, cell-type-specific therapeutic intervention in HGPS to prevent permanent vascular tissue damage. In addition, the cell-type-specific molecular dynamics of the aortic arch VSMCs and fibroblasts during HGPS disease progression are provided in a user-friendly searchable scRNA-seq database available for preclinical research targeting vascular aging.

**Supplementary Information:**

The online version contains supplementary material available at https://doi.org/10.1186/s13073-026-01719-6.

## Background

Hutchinson-Gilford Progeria Syndrome (HGPS), also known as progeria, is a very rare segmental premature aging disease (1 patient per 18 million individuals [1]) caused by de novo mutations in the *LMNA* gene [2, 3]. *LMNA* encodes for Lamin A and Lamin C, that, together with B-type lamins, are key components of the nuclear lamina [4]. In particular, the *LMNA* c.1824 C > T mutation, which accounts for over 90% of HGPS cases, leads to the activation of a cryptic splice site in exon 11 of the *LMNA* gene that results in the production of a partially processed form of prelamin A named progerin [2, 5]. Progerin remains permanently farnesylated, leading to enhanced association with the nuclear lamina [6], which has dramatic effects on nuclear structural integrity [6], genome organization and epigenetic maintenance, among others [7].

HGPS patients appear healthy at birth, but progressively develop a wide range of symptoms, including failure to thrive, scleroderma-like skin changes and skeletal dysplasia. Some symptoms overlap with features of normal aging, such as subcutaneous fat loss, alopecia or joint stiffness [7]. In addition, HGPS-affected children show a steadily declining vascular function and develop cardiac abnormalities [8] eventually resulting in death at around 14.6 years of age [1] from heart failure, myocardial infarction, or stroke [9, 10]. HGPS patients exhibit advanced atherosclerosis in the absence of hypercholesterolemia [7, 9]. Histopathological analysis of the vasculature from deceased HGPS patients revealed the presence of atheroma-like regions, extensive adventitial fibrosis and thickening, extracellular matrix (ECM) remodeling due to collagen and proteoglycan deposition, and vascular smooth muscle cell (VSMC) loss [10–13]. At the molecular level, progerin itself is known to drive VSMC loss, and elevated endoplasmic reticulum (ER) stress [14] and PARP1 inactivation [15] have been described in murine progeria VSMCs. Progeric endothelial cells are characterized by defective mechanotransduction [16, 17] and higher YAP/TAZ activation [18]. In parallel, progeria mice also display extensive adventitial fibrosis, with fibroblasts adopting inflammatory states [19]. Despite these advances, an integrative mechanism by which progerin induces vascular disease is still lacking.

Under physiological conditions, VSMCs are quiescent contractile cells that regulate vascular tone [20–22] and are characterized by the expression of markers such as *Acta2*, *Myh11* or *Tagln* [23]. However, upon vascular injury or atherosclerosis, VSMCs can reduce the expression of contractile markers and proliferate oligoclonally to promote tissue repair [24, 25]. These “synthetic” VSMCs express markers such as *Lgals3*, *Vcam1* or *Ly6a* [22] and can further acquire features of other cell types, such as macrophages (*Cd68* with or without *Lgals3*), fibroblasts (*Spp1*, *Tnfrsf11b*, *Fn1*) or osteoblasts (*Runx2*, *Sox9*) [20–22]. Under physiological conditions, VSMC phenotypic switching represents a transient response to vascular injury, but in disease states, phenotypically switched VSMCs can persist and contribute to vascular disease development [20].

To define the key molecular programs driving the vascular phenotype in HGPS, we performed single-cell RNA-sequencing (scRNA-seq) in the aortic arch of *Lmna*^*G609G/G609G*^ mice, a well-established *knock-in* mouse model genetically engineered to carry the mouse equivalent of the human HGPS-causing mutation (c.1827C > T) [26], and that expresses Lamin A, Lamin C and progerin. *Lmna*^*G609G*^ allele-carrying mice faithfully recapitulate the HGPS phenotype, including the vascular features in the absence of an atheroprone environment. Homozygous mice (*Lmna*^*G609G/G609G*^) exhibit profound VSMC loss in the aortic arch [26], and heterozygous mice (*Lmna*^*G609G/*+^) display aortic calcification [27], although their VSMC loss is less dramatic [28]. We performed a detailed histological characterization of the aortic arch that showed that *Lmna*^*G609G/G609G*^ mice suffered a progressive VSMC loss in the aortic arch, starting at 7 weeks of age. We also observed a transient increase in VSMCs expressing proliferative markers, and an eventual accumulation of apoptotic VSMCs. Furthermore, we detected transcriptionally distinct VSMC and fibroblast populations that were either absent or rare in the wild-type mice. Disease-enriched VSMCs initially displayed elevated expression of ER stress and ROS-related genes. At later stages, features of VSMC phenotypic switching and apoptosis were also detected, highlighting a phenotypic drift associated with disease progression. Furthermore, VSMCs from 12-week old *Lmna*^*G609G/G609G*^ mice showed a global increase in DNA damage and in the number of somatic single-nucleotide variants (SNVs), which became more pronounced as disease progressed. Notably, in highly stressed cells, somatic SNVs accumulation correlated with the ER stress and ROS responses, as well as p53 transcriptional program, suggesting an induction of senescence.

In parallel, disease-enriched fibroblasts became activated or cartilage-producing, consistent with pathological vascular remodelling. *Lmna*^*G609G/G609G*^ fibroblasts also accumulated DNA damage with disease progression but only showed significant somatic SNVs accumulation by 12 weeks of age, underscoring cell-type-genomic vulnerability to progerin.

Altogether, we present a comprehensive single-cell transcriptomic atlas of key cell types in the vascular wall of the *Lmna*^*G609G/G609G*^ aortic arch, capturing the VSMC and fibroblast cellular state transitions and features of genomic instability during disease progression. This data is provided as an interactive database, providing a platform to accelerate the discovery of novel pathological mechanisms and therapeutic targets relevant to both HGPS and vascular aging.

## Methods

### Animal maintenance

All procedures in mice followed the institutional guidelines and regulations of Karolinska Institutet. The animal research was approved by the animal research ethical review board in Linköping (Dnr 6088–2020 and Dnr 20490–2024). The experimental mice were maintained at the pathogen-free animal facility Karolinska Institutet, Campus Flemingsberg. The mice were housed at 20–22 °C temperature, with 50–65% humidity, with a 12-h light/dark cycle. *Lmna*^*G609G*^/+ mice were kindly provided by Carlos López Otín and have been previously published [26]. *Acta2*-GFP mice were obtained from Christer Betsholtz and have been previously published [29]. Both mice strains were crossed to obtain *Lmna*^*G609G/G609G*^*:Acta2*-GFP mice. *Lmna*^+/+^, *Lmna*^*G609G/G609G*^*, Lmna*^+/+^*:Acta2*-GFP and *Lmna*^*G609G/G609G*^*:Acta2*-GFP mice were included in the study. Overall, we used:

- 3 *Lmna*^+/+^ mice aged 6 weeks (3 females)
- 7 *Lmna*^+/+^*:Acta2*-GFP mice aged 6 weeks (6 males, 1 female)
- 5 *Lmna*^+/+^ mice aged 12 weeks (3 males, 2 females)
- 5 *Lmna*^+/+^*:Acta2*-GFP mice aged 12 weeks (3 males, 2 female)
- 3 *Lmna*^*G609G/G609G*^ mice aged 6 weeks (3 females)
- 6 *Lmna*^*G609G/G609G*^*: Acta2*-GFP mice aged 6 weeks (3 females, 3 males)
- 12 *Lmna*^*G609G/G609G*^ mice aged 10 weeks (7 males, 5 females)
- 5 *Lmna*^*G609G/G609G*^ mice aged 12 weeks (2 males, 3 females)
- 7 *Lmna*^*G609G/G609G*^*: Acta2*-GFP mice aged 12 weeks (5 females, 2 males)

For genotyping, genomic DNA was extracted using the Puregene Tissue Kit (Qiagen 158,063) following manufacturer’s instructions.

The following published primers [26] were used for *Lmna*^*G609G/G609G*^ genotyping:

- 5’ -AAGGGGCTGGGAGGACAGAG-3’
- 5’ -AGCATGCAATAGGGTGGAAGGA-3’
- 5’ -AGTAGAAGGTGGCGCGAAGG-3’

And the following primers were used for *Acta2*-GFP mice genotyping:

- Forward: 5’ -CCTACGGCGTGCAGTGCTTCAGC-3’
- Reverse: 5’ -CGGCGAGCTGCACGCTGCGTCCTC-3’

### Murine aortic arch collection

Mice were euthanized by isoflurane overdose followed by cervical dislocation. Heart perfusion with PBS was performed to remove all blood from the vasculature. For each mouse, the aortic arch was carefully microdissected and cleared of fat under ice-cold PBS. When performing single-cell RNA-sequencing, the aortic arches were placed in ice-cold PBS until further processing. For validation experiments and assessing the effect of the TUDCA treatment (immunohistochemistry and ddPCR), the aortic arch was divided in 2 pieces following dissection. The upper part, closer to the heart, was fixed overnight in PFA 4% at 4 °C and stored in 70% ethanol until further processing. The lower part was immediately frozen in dry ice and stored at −80 °C.

### Generation of a single cell suspension from the murine aortic arch

The dissected aortic arches were enzymatically digested to obtain a single-cell suspension. Two different protocols were used. In both cases, the aortic arches were opened longitudinally and minced into 1 mm pieces prior to digestion. In protocol 1, each aortic arch was digested in HBSS (Gibco, 14,025–092) containing 2% Bovine Serum Albumin (Merck, A9647), 250 µg/ml of collagenases I (Merck, C5894), II (Merck, C6885) and IV (Merck, C1889), 5.98 U/ml porcine elastase (Worthington Biochemical, LS002279), 2.5 mM CaCl2 and 125.75 U/ul DNase (Invitrogen, 18,047–019). The samples were subjected to 3 consecutive incubations of 10 min each (37 °C and shaking at 450 rpm), resuspending 20 times between each incubation. To remove large residual pieces, the cells suspensions were filtered twice using a 70 μm and a 40 μm strainer. In protocol 2, the aortic arches were digested in 24-well tissue culture plates in sterile DMEM containing 2.5 mg/ml of collagenase IV (Gibco, 17,104–019) and 2.5 U/ml of porcine elastase. 2 h incubations were performed in a cell incubator, at 37 °C and 5% CO_2_, resuspending the solution every 30 min. To stop the digestion, the cells were transferred to a 50 ml Falcon containing BSA 1% in PBS. Subsequently, the cells were filtered through a 70 μm and a 40 μm strainer.

### Viability staining and Fluorescence Activated Sorting (FACS) for scRNA-seq

Following digestion, arterial cells were centrifuged for 5 min (300 g, 8 °C) and resuspended in BSA 1% in PBS. To assess viability, the cells were stained with 5 μM DRAQ5 (Thermo Scientific, #62,251) and 9 µg/ml Calcein Green (Invitrogen, C34852). Following a 15 min incubation on ice, the BD FACS Aria Fusion was used to individually sort arterial cells based on size and double-positivity for Calcein Green and DRAQ5 into the wells of 384-well plates containing Smart-seq2 lysis buffer. In some cases, GFP sorting was performed to enrich for VSMCs (Additional file 1: Table S1). After sorting, the plates were immediately sealed, frozen in dry ice and stored in −80° C until further processing.

### Sequencing

Sequencing was performed at the Single Cell Core Facility of Flemingsberg (SICOF) with the Smart-seq2 protocol [30]. Smart-seq2 was chosen because it provides full and uniform transcript coverage and has high sensitivity for gene detection. Briefly, cDNA was synthesized using SuperScript II reverse transcriptase and oligo(dT) primers (ThermoFisher Scientific), as well as a template-switching oligonucleotide for second-strand cDNA synthesis. cDNA was then amplified using PCR for 23–26 cycles. TapeStation 4200 or a 2100 Bioanalyzer with a DNA High Sensitivity chip (Agilent Biotechnologies) were used for performing quality control (QC). Samples that passed QC were subsequently fragmented and tagmented with Tn5 transposase, and each well was uniquely indexed using the Illumina Nextera XT index kits (Sets A–D). Uniquely indexed cDNA libraries from a 384-well plate were pooled into a single sample and sequenced on one lane of an Illumina HiSeq 3000 instrument with dual indexing and single-end 50 base-pair reads. Plate information is summarized in Additional file 1: Table S1.

### Data analysis

Sequencing reads were trimmed used Trim Galore v.0.4.4, which wraps Cutadapt v.1.11 [31], in single-end mode. Adapter trimming was performed using the Nextera trasposase adapter sequence (CTGTCTCTTATA). Adapter matches were required to have a minimum overlap of 3 bp and allowed a maximum error rate of 10%. In addition to adapter removal, reads were quality-trimmed at a Phred score cutoff of 20 (ASCII + 33 encoding). Reads shorter than 20 bp after trimming were discarded. Subsequently, alignment to the mouse reference genome GRCm39/mm39 [32] was performed using Hisat2 [33] v.2.2.1. Subsequently, we applied RSEM [34] v.1.3.3 for quantifying gene expression and to generate a gene expression count matrix. From the count matrix, genes with less than 5 counts across all cells were filtered out. We used Seurat [35] (version ≥ 4.0.2) to create a Seurat Object and we further subset the data by selecting cells matching the following criteria: mitochondrial content below 20%, ERCC spike-in percentage below 30%, expression of over 1500 genes and library size between 100,000 and 10,000,000 counts. These parameters were based on a previous scRNA-seq study in VSMCs [23]. Additionally, mitochondrial genes (mt) and ERCC sequences were removed. Using Seurat, we performed principal component analysis (PCA) based on the 2000 most variable features, clustered the cells (dimensions = 22, resolution = 0.4) and visualized the results using UMAP, with the specification “umap-learn” to achieve a more compact representation. We integrated Harmony 0.1.0 [36], into the analysis pipeline to account for potential transcriptional effects of using two different digestion methods. Cluster identity was assigned based on the expression levels of known markers of cell identity. Cell types placed in the UMAP together with a different cell type were removed prior to performing gene expression comparisons. The function FindAllMarkers was used for overall cluster characterization (min.pct and logfc.threhold = 0.25), whereas FindMarkers with default settings was used specifically to identify differentially expressed genes (DEGs) between clusters of interest. VSMC subpopulation V was enriched in unknown transcripts, so when comparing gene expression across subpopulations, transcripts starting by “Gm”, characteristic of the VSMC V cells, were excluded from volcano plots for better visualization. Gene set enrichment analysis (GSEA) was performed using the clusterProfiler v4.16.0 R package [37], with the gseGO function and annotations from the org.Mm.eg.db database [38] (mouse). Enrichment was performed across all three GO ontologies (Biological Process, Molecular Function, and Cellular Component). Parameters were set to 10,000 permutations, a minimum gene set size of 10 and maximum of 300, and a p-value cutoff of 0.01 and the Benjamini–Hochberg procedure for multiple testing correction. Monocle3 (Trapnell lab) [39] with default settings was applied for trajectory analysis. CellChat [40] v.1.6.1 using default parameters was used for cellular communication analysis.

For comparing cell frequency across genotypes and ages and avoid biases caused by unequal cell numbers between mice sorted or not for GFP, the larger GFP-positive populations were randomly downsampled to match the number of GFP-negative cells within each age group using a fixed random seed. Cluster frequencies were then calculated by combining GFP-positive and GFP-negative cells within each age group.

### Progerin and Lamin A transcript detection

To accurately measure the levels of the progerin and Lamin A transcripts, we used BWA-MEM [41] v.2.2.1 to align all sequencing reads to a 100 nucleotide-long reference sequence specific for either the progerin or the lamin A transcript. Each of the reference sequences contained 50 nucleotides upstream and downstream of the exon11-exon12 junction that differs between the lamin A and the progerin transcript. To quantify the reads mapping specifically to either Lamin A or progerin, we used samtools [42] v.1.2 to further retain only aligned reads mapped across the splice junction between exons 11 and 12 and contained at least 6 nucleotides of the progerin/Lamin A transcript (reads starting latest at position 46 of the reference sequence and that finished after position 52). Subsequently, we counted the number of specific progerin/Lamin A reads per cell and overlayed the data with the previously generated Seurat object for visualization. Log_10_ of counts + 1 was used for data visualization.

- Lamin A reference (nucleotides at the splice junction are highlighted)
  ACCTAGTCACCCGCTCCTACCTCCTGGGCAA
  CTCCAGTCCCCGGA**GCCAGAGCT**CCCAGAA
  CTGCAGCATCATGTAATCTGGGACCTGCCAG
  GCAGGGCT
- Progerin reference (nucleotides at the splice junction are highlighted)
  GGACGTGTGGGCAGCCTGCTGACAAGGCTG
  CCGGTGGAGCGGGAG**CCCAGAGCT**CCCAGA
  ACTGCAGCATCATGTAATCTGGGACCTGCCA
  GGCAGGGCT

As control, we also aligned all sequencing reads to *Lmna* exons 1, 11 and the *Lmna* 150 nt sequence in exon 11 that is lost in progeria. All mapped reads were kept. The used sequences are the following:

- *Lmna* exon 1:
  AGAGCCTCTGCCTGGCTGTCAGTGACTCAG
  TGTTCGCGGGAACGCTGCCTCAGCCTCAAC
  ACCAGCCAACCCAGATCCCGAGGTGCGCCA
  GCGCCCAGCCCAGATCTCCACGCCTGCCAG
  GAGCGAGCTTCGCCGGCTCGCTGTCCCCCT
  GAGCAGCCTCTGTCCTTCTGTCCAAGTCCCG
  CGCCCTTCTCGGGACCCCTGCCCAGCGGGC
  AGCACTGTCACCCTGCCGGCCATGGAGACC
  CCGTCACAGCGGCGCGCCACCCGCAGTGGG
  GCGCAGGCCAGCTCTACCCCACTGTCGCCC
  ACTCGGATCACCCGGCTGCAGGAGAAGGAG
  GACCTGCAGGAGCTCAATGACCGCCTGGCC
  GTGTACATCGATCGCGTGCGTTCCCTGGAGA
  CCGAGAACGCGGGGCTGCGCCTTCGCATCA
  CTGAGTCTGAAGAGGTGGTCAGCCGAGAGG
  TGTCCGGCATCAAGGCGGCCTACGAGGCCG
  AGCTGGGGGATGCCCGCAAGACCCTTGATT
  CTGTGGCCAAGGAGCGCGCCCGCCTCCAGC
  TAGAGCTGAGCAAAGTGCGTGAGGAGTTCA
  AGGAGCTGAAGGCTCG
- *Lmna* exon 11:
  GGTTCCCACTGCAGCGGCTCGGGGGACCCC
  GCTGAGTACAACCTGCGCTCACGCACCGTG
  CTGTGCGGGACGTGTGGGCAGCCTGCTGAC
  AAGGCTGCCGGTGGAGCGGGAGCCCAGGTG
  GGCGGATCCATCTCCTCTGGCTCTTCTGCCT
  CCAGTGTCACAGTCACTCGAAGCTTCCGCAG
  TGTGGGGGGCAGTGGGGGTGGCAGCTTCGG
  GGACAACCTAGTCACCCGCTCCTACCTCCTG
  GGCAACTCCAGTCCCCGGAGCCAG
- 150 nucleotides deleted in progeria:
  GTGGGCGGATCCATCTCCTCTGGCTCTTCTG
  CCTCCAGTGTCACAGTCACTCGAAGCTTCCG
  CAGTGTGGGGGGCAGTGGGGGTGGCAGCTT
  CGGGGACAACCTAGTCACCCGCTCCTACCTC
  CTGGGCAACTCCAGTCCCCGGAGCCAG

### Somatic mutation detection

Somatic mutation calling was performed using the SComatic algorithm (downloaded from GitHub on 15 April 2025) [43], with the overall modification of using min_MQ = 30 throughout the analysis. Briefly, all individual BAM files for each collected cell from the experiment were merged into a single BAM. We used *SplitBamCellTypes.py* with default parameters except, max_nM = 5 and max_NH = 1 and split the BAM file in 10 BAM files based on the genotype of the mouse and the cell type inferred from the clustering analysis (e.g. VSMC_WT, VSMC_G609G, Fibroblast_WT or Fibroblast_G609G). We proceeded with VSMCs and Fibroblasts since they were the most abundant cell types and applied BaseCellCounter.py and MergeBaseCellCounts.py with default parameters to obtain a count matrix containing base count information for each cell type. BaseCellCalling.step1.py, was subsequently used to call somatic mutations. The alpha and beta parameters for the beta binomial distribution were determined using a separate published Smart-seq2 dataset on vascular cells [44] using the BetaBinEstimation.py script. The obtained parameters were: alpha 1, 1.0543136200991154; alpha 2, 0.6117853860688995; beta 1, 311.866453189087; and beta 2, 578.3857601985449. We further used BaseCellCalling.step2.py to remove SNPs positions described in REL2021 from the mouse genome project (mm39). The min_distance parameter was switched off to allow the detection of mutations caused by dinucleotide DNA damage. Mutations labelled as PASS were used to determine the genotype of each individual cell with the SingleCellGenotype.py. To ensure we were detecting real mutations and not artifacts, we further filtered the mutations by selecting those mutations supported by at least 3 reads in one cell. In addition, any mutation occurring in a cell from the VSMC V subpopulation was removed, due to its artifactual nature. The number of callable sites per cell was calculated using the GetAllCallableSites.py script with default settings. Cells with less than 100,000 callable sites were excluded based on histogram visualization. Since differences in sequencing depth can impact the likelihood of detecting somatic mutations, we also determined the average sequencing depth per cell using samtools [42] depth and used it for normalization. Using all this information, we calculated the mutation rate as number of mutations per megabase per cell using the following formula:

$$\frac{mutations per cell}{callable sites per cell*average sequencing depth per cell}*1000000$$

When comparing progeria and wild-type mice, only cells with detected mutation above zero were selected. Cells with mutation load zero were not considered representative.

To identify mutations that were likely to be recurrent, we selected mutations occurring in at least 2 cells from the same batch of mice (Additional file 1: Table S1). Ensembl Variant Effect Predictor [45] (VEP v111) was used for variant annotation. All identified mutations were SNVs.

### Mutational signatures

To determine mutational signatures, we used the MutationalPatterns v.3.18 R package [46]. High-confidence mutations (≥ 3 supporting reads), from wild-type and progeria VSMCs/Fibroblasts we used to create GRanges objects, which were subsequently converted into mutational matrices covering the 96 trinucleotide mutation contexts. Relative mutation counts were normalized for trinucleotide opportunities using genome-wide Smartseq2-specific trinucleotide frequencies. The genome wide trinucleotide frequencies were manually calculated in R using the mouse reference genome (BSgenome.Mmusculus.UCSCmm39) [32]. Chromosomes 1–19, X, and Y sequences were extracted, and trinucleotide counts were determined for each chromosome. Counts were collapsed to canonical pyrimidine-centered trinucleotides by summing each trinucleotide with its reverse complement and orienting sequences according to the middle base. Resulting counts were normalized to relative frequencies. The Smartseq2 trinucleotide frequency was determined by TrinucleotideContextBackground.py from SComatic. Normalization of mutation counts was performed according to the formula:

$$relative mutation counts* \frac{trinucleotide frequency in the mm39 genome}{trinucleotide frequency in our Smartseq2 data}$$

Known mutational signatures for the mm10 genome were fitted using fit_signatures_strict with a maximum delta of 0.04 for VSMCs and 0.01 for fibroblasts, limiting the analysis to fewer than 5 fitted signatures per sample.

### Differentially expressed genes in high mutation clones

We defined high mutation load VSMCs as those VSMC with a SNV per megabase greater than the third quartile (Q3), which corresponded to 4.5 SNVs per cell. Based on this threshold, progeria VSMCs were classified as: High mutation load: > 4.5 SNVs per cell and Low mutation load: < 4.5 SNVs per cell. Wild-type VSMCs were included regardless of mutation load.

Differentially expressed genes (DEGs) were determined using the function FindMarkers to compare: 1. High mutation load progeria VSMCs vs. wild-type VSMCs and 2. Low mutation load progeria VSMCs vs. wild-type VSMCs. Genes were considered differentially expressed if the adjusted p-value (Benjamini–Hochberg correction) was < 0.05. To identify transcriptional changes specifically associated with high mutation burden, genes that were also significantly upregulated in the low mutation load comparison were removed from the high mutation load gene list. The remaining upregulated genes were used on the online STARNET browser (http://starnet.mssm.edu/).

### Transcriptional signature detection

To assess the combined expression of genes of a specific biological process, we used the Seurat function AddModuleScore, which works as follows: Transcriptional scores are calculated for each individual cell by averaging the expression of the target genes and later subtracting the aggregated expression of matched control gene sets. To account for differences in baseline gene expression, all genes are binned according to their average expression across the dataset, and control genes are randomly selected from the same expression bins as the target genes. This strategy provides as estimate of the target genes activity while reducing bias potentially introduced by differences in overall gene expression.

Relevant genes for each process from the significantly upregulated genes with min.pct = 0.1 in the progeria-enriched populations were selected. The genes used for each score:

- ER stress score:*Asns, Atf4, Ddit3, Gdf15, Hmox1, Hsp90b1, Hspa5, Sdf2l1* (ER stress related genes previously shown to be upregulated in *Apoe*^−/−^ *Lmna*^*G609G/G609G*^and that were also upregulated in VSMC II cells) [14].
- Apoptosis: *Bad, Bax, Bid, Fas, Pmaip1, Trp53*(apoptosis-related genes observed in the VSMC II upregulated genes) [47].
- Aortic VSMC score: *Actn4, Cnn1, Dstn, Itgb1, Myh11, Mylk, Sdc4, Smtn, Tagln, Tpm1 (*previously described to define VSMC identity) [23].
- SMC score general: *Acta2, Actg2, Cnn1, Dmpk, Flna, Lmod1, Tagln, Myh11, Mylk, Myl9, Tpm2, Ppp1r12b*(described a non-tissue specifc SMC score) [48].
- ROS score: *Ftl1, Gclm,, Hmox1, Sesn2, Srxn1, Txnl1, Txnrd1*(genes connected to ROS or an antioxidant response that were selected from the VSMC II upregulated genes) [49–52].
- p53 score: *Trp53, Mdm2, Cdkn1a, Gadd45a, Zmat3,* and *Trp53inp1* (p53 transcriptional targets) [53–55].
- Cartilage score: *Col2a1, Col9a1, Col9a2, Col9a3, Col11a1, Col11a2*(upregulated genes coding for collagen isoforms in Fibroblast II cells known to form cartilage) [56].
- Activation score: *Cthrc1, Postn, Thbs4, Tnc, Tnn*(genes characteristic of fibroblast activation among the upregulated genes in Fibroblast III cells) [57–60].
- Fibroblast score: *Col1a1, Col1a2, Col3a1, Col4a1, Col4a2, Col5a1, Col5a2, Col6a1, Col6a2, Col6a3, Col14a1, Col16a1*(genes encoding for structural collagens downregulated in Fibroblast II cells) [61].

### Data availability

The expression data has been made publicly available in the form of a user-friendly interactive database, which can be accessed by clicking the following link: https://www.Progeriadatabase. For visualization purposes, the names of the VSMC and fibroblasts subpopulations were adapted in the database, but the colors were preserved. The equivalences are •For VSMCs: Conserved I = VSMC III; VSMC IV; Conserved II = VSMC V, Conserved III = VSMC I; Contractile = VSMC IV; Rgs5 + = VSMC VI; Diseased = VSMC II •For fibroblasts: Conserved = Fibroblast I, Disease I = Fibroblast II, Disease II = Fibroblast III. The raw and processed data have been deposited in GEO (GSE31747163, https://www.ncbi.nlm.nih.gov/geo/query/acc.cgi?acc=GSE317471) and are publicly available.

- For VSMCs: Conserved I = VSMC III; VSMC IV; Conserved II = VSMC V, Conserved III = VSMC I; Contractile = VSMC IV; Rgs5 + = VSMC VI; Diseased = VSMC II
- For fibroblasts: Conserved = Fibroblast I, Disease I = Fibroblast II, Disease II = Fibroblast III.

The raw and processed data have been deposited in GEO (GSE317471 [62], https://www.ncbi.nlm.nih.gov/geo/query/acc.cgi?acc=GSE317471) and are publicly available.

### VSMCs density

VSMC density analysis was performed on both H&E and DAPI stained tissue sections. In both instances, nuclei were manually counted and normalized by the medial area using, respectively, Fiji ImageJ (version 2.14.0/1.54f) and QuPath (version 0.6.0) [63]. In immunofluorescence, medial area was delimited by the autofluorescence of the internal and external elastin fibers.

### Immunostaining

PFA-fixed tissues were paraffin-embedded and transversally cut in 4 µm sections. For immunostaining, individual sections were deparaffinized in xylene (VWR, 28975325), progressively rehydrated in 100% and 70% ethanol and washed briefly in milliQ water. From this point, all steps were separated by a 5 min wash in PBS. Heat-mediated antigen retrieval was performed by boiling the samples in a microwave for 25 min in 10 mM sodium citrate buffer containing Tween20 (Merck, P9416, 1:1000). Subsequently, the samples were permeabilized in Tween20 0.2% in PBS for 10 min at RT and blocked for 45 min at RT using BSA 1% in PBS. The slides were incubated overnight with an antibody against either Lgals3 (ThermoFisher Scientific, #14–5301-82, 1:300), BiP (Cell Signaling, 3177, 1:200), progerin (Enzo Life Science, 13A4, 1:150), γH2AX (Sigma Aldrich, ZMS05636, 1:1000), Pcna (Abcam, ab18197, 1:2000), Ki67 (Abcam, ab15589, 1:500) or cleaved-caspase 3 (CC3, Cell signaling, D175, 1:200), diluted in 1% BSA (Merck, A9647) and 10% NGS (Abcam, ab7481) in PBS. Afterwards, 4 PBS washes of 5 min at RT with shaking were performed and the samples were incubated for 1 h at RT with the specific secondary antibody diluted in 1% BSA and 10% NGS in PBS: for Lgals3, Alexa Fluor 488 conjugated goat anti-rat (ThermoFisher Scientific, #A-11006, 1:300); for BiP and γH2AX, Alexa Fluor 647, goat anti-rabbit (1:400 for BiP and 1:500 for γH2AX, #A21245). For progerin, Alexa Fluor 488 conjugated goat anti-mouse (ThermoFisher Scientific, # 10256302, 1:800). When Anti-actin α-smooth muscle-Cy3 was used, it was following secondary antibody incubation (Sigma-Aldrich, C6198, 1:3000).

### TUDCA injections

*Lmna*^*G609G/G609G*^ mice were intraperitoneally injected with either PBS (n = 4) or tauroursodeoxycholic acid (TUDCA, Merck, 580,549) (n = 5) dissolved in PBS at a dose of 400 mg/kg, as previously described [14]. The injection volume was 10 ml/kg. The mice were injected 3 times/week for 4 consecutive weeks, spanning ages 6 to 10 weeks. Organ collection was done 1 day after the last injection.

### RNA extraction and cDNA synthesis from the mice aortic arch

RNA was extracted from snap-frozen aortic arches samples from wild-type, *Lmna*^*G609G/G609G*^ mice, and *Lmna*^*G609G/G609G*^ mice treated with PBS or TUDCA, using the RNeasy Plus Universal kit (Qiagen). RNA concentration and quality were evaluated using the NanoQuant Infinite M200 Pro (Tecan). A total of 0.2–1.3 μg of RNA were extracted from the aortic arches. 0.1 μg RNA were used for the cDNA synthesis, which was performed using the SuperScript IV kit (Invitrogen, Life Technologies), with random hexamers, following the manufacturer’s instructions.

### Absolute quantification by ddPCR

Absolute quantification was performed using the QX200 ddPCR system (Bio-Rad). The following primers were used:

- *Spp1* forward: 5′-TTGGCAGTGATTTGCTTTTG-3′
- *Spp1* reverse: 5′-TCTGGGTGCAGGCTGTAAA-3′
- *Ddit3* forward: 5′-ATATCTCATCCCCAGGAAACG-3′
- *Ddit3* reverse: 5′- CTCCTGCTCCTTCTCCTTCAT −3′
- *β-actin* forward: 5′-CCTAGGCACCAGGGTGTGAT-3′
- *β-actin* reverse: 5′-CCATGTCGTCCCAGTTGGTAA-3′

The PCR reactions used 2 × QX200 EvaGreen Mix (Bio-Rad), forward and reverse primers of interest. Each sample was run in duplicate in two wells. *β-actin* was used for normalization for the other genes. The analysis was performed according to previously described procedures [64].

### RNAscope

RNAscope was performed using RNAscope™ Multiplex Fluorescent Reagent Kit v2 (Cat. No. 323100, ACDbio) according to manufacturer protocol (UM 323100), on freshly cut FFPE sections. Briefly, slides were baked at 60° for 1 h and later deparaffinized in xylene and 100% ethanol. Endogenous peroxidase activity was blocked with hydrogen peroxide for 10 min at RT and target retrieval was performed in the water bath at 99 °C for 15 min. All the following incubations were performed on HybEZ Humidity Control Tray (PN 310012) in HybEZ oven (PN 321710). Tissues were permeabilized with RNAscope Protease Plus (cat. 322,381) for 30 min at 40 °C and the following probes were added on the slides: positive control (cat. 310,771), negative control (cat. 320,871) and Vcam1 (cat. 438,641); each of the probes was incubated for 2 h at 40 °C. The signal was amplified with subsequent incubations with Amp1 and Amp2 for 30 min at 40 °C and Amp3 for 15 min at 40 °C. Signal was developed by incubating the slides with RNAscope Multiplex FL V2 HRP-C1 for 15 min at 40 °C, followed by incubation with TSA Plus Cy3 reagent (cat. TS-000202, Akoya Biosciences) (1:1500) for 30 min at 40 °C and blocked with RNAscope multiplex FL V2 HRP blocker for 15 min at 40 °C. Nuclei were counterstained with RNAscope DAPI.

### TUNEL assay

We used the In Situ Cell Death Detection kit, TMR red (Roche) for measuring cell death in the aortic arches following the manufacturer´s instructions. DRAQ5 (1:800) was used for nuclear staining.

### Imaging

Immunofluorescence imaging was performed on a Nikon A1R and A1 + single point scanning confocal, using the NIS Elements software (Nikon corporation), and imaging with a 20X air objective. For each sample, we acquired large images to cover the entire aortic tissue, which were stitched in one final image. We also captured Z-stacks to cover the whole aortic thickness, with 1.5 µm separation in between adjacent sections. Maximum intensity projection (MaxiIP) images were generated from the Z-stacks and analyzed.

### Statistical analysis

**Fig. 1:**
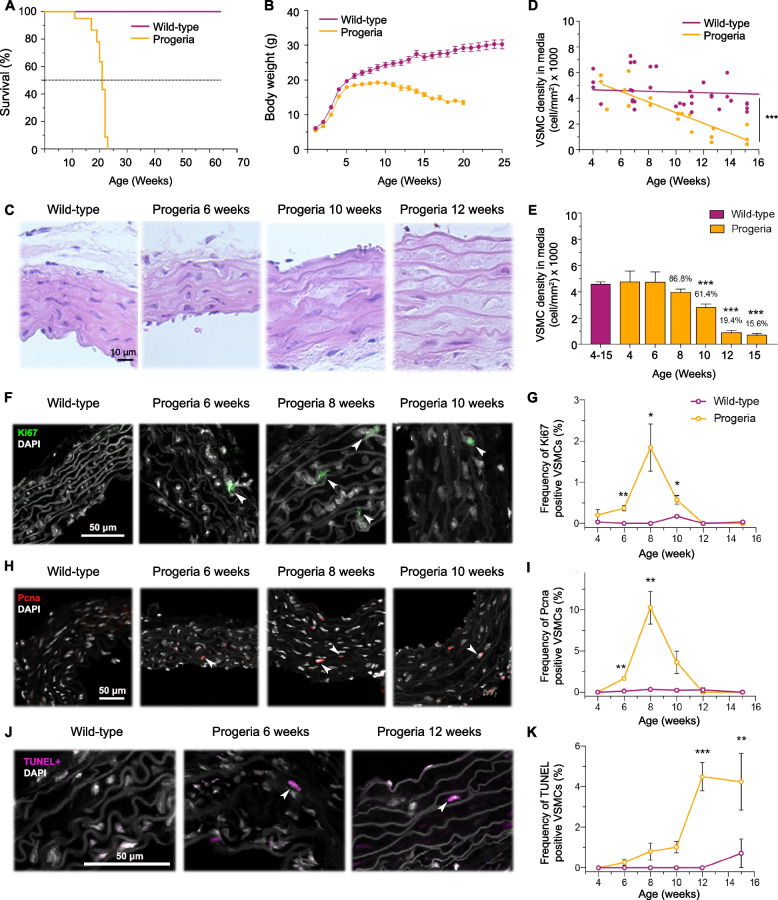
Phenotypic characterization of the *Lmna*^*G609G*/G609G^ (progeria) mice. **A** Survival analysis of wild-type mice (from Osorio et al. 2009, Sci Transl Med) and progeria mice (*n* = 36). Dotted line indicates 50% survival. **B** Body weight measurements (grams) of wild-type (*n* = 20) and progeria mice (*n* = 25) for 25 weeks. **C** Representative images of hematoxylin–eosin staining of the aortic arch from wild-type and progeria mice. For the progeria mice the ages 6, 10 and 12 weeks are shown. **D** and **E** Evolution of VSMC density (cells/mm^2^) in the aortic arch of wild-type mice of ages 4 (*n* = 4), 6 (*n* = 9), 8 (*n *= 3), 10 (*n* = 7), 12 (*n* = 6) and 15 weeks (*n* = 3) and progeria mice of ages 4 (*n* = 3), 6 (*n* = 3), 8 (*n* = 2), 10 (*n* = 5), 12 (*n* = 3) and 15 weeks (*n* = 3) shown continuously (D) or in progeria mice of ages 4 (*n* = 3), 6 (*n* = 3), 8 (*n* = 3), 10 (*n* = 7), 12 (*n* = 7) and 15 weeks (*n* = 7) compared to pooled wild-type mice of ages 4 (*n *= 3), 6 (*n* = 4), 8 (*n* = 4), 10 (*n* = 5), 12 (*n* = 5) and 15 weeks (*n* = 4) (E). **F** Representative images from Ki67 immunofluorescence (green) in aortic arch sections from wild-type and progeria mice the ages 6, 8 and 12 weeks. Nuclei are shown in white. **G** Percentage of Ki67-positive VSMCs in the aortic arch of wild-type and progeria mice from ages 4 to 15 weeks (*n* = 3 for each age). **H** Representative images from Pcna immunofluorescence (red) in aortic arch sections from wild-type and progeria mice the ages 6, 8 and 12 weeks. Nuclei are shown in white. **I** Percentage of Pcna-positive VSMCs in the aortic arch of wild-type and progeria mice from ages 4 to 15 weeks (*n* = 3 for each age). **J** Representative images from TUNEL assay (pink) in aortic arch sections from wild-type and progeria mice the ages 6 and 12 weeks. **K** Percentage of TUNEL-positive VSMCs measured by the TUNEL assay in the aortic arch of wild-type and progeria mice from ages 4 to 15 weeks (*n* = 3 for each age). Nuclei are shown in white. Statistical test used in panel D is one-way ANOVA followed by Dunnet; for panels G and I, unpaired two-tailed Student´s t-test and for panel K, one way ANOVA followed by TukeyHSD. For panel D: slopes were compared using an extra sum-of-squares F test (GraphPad Prism). * = *p*-value < 0.05; ** = *p*-value < 0.01; *** = *p*-value < 0.001. Arrowheads in panels F, H and J point to examples of positive cells in the stainings. Scale bar indicated 10 µm in panel C, and 50 µm in panels F, H, J

The immunohistochemistry, TUNEL and RNAscope data is presented as mean ± SEM. Normality of the data was assessed using the Shapiro–Wilk test. In Fig. 1D, slopes were compared using an extra sum-of-squares F test (GraphPad Prism).

For normally distributed data, a two-tailed Student´s t-test or one-way ANOVA followed Tukey´s post hoc test was used to test for significance, depending on sample number. For data that were not normally distributed, the Wilcoxon test or Kruskal–Wallis test followed by Dunn post hoc test were used. For comparisons of gene expression or transcriptional scores across cell subpopulations, the Wilcoxon test or Kruskal–Wallis test followed by pairwise Wilcoxon tests was used, as appropriate. Immunohistochemical analysis was performed blindly, with the researchers having knowledge of the experimental mice group upon performing staining, imaging and quantification.

## Results

### Time-dependent progression of aortic phenotypes in the progeria mouse model

When housed in our animal facility and under our feeding conditions, the median survival age of *Lmna*^*G609G/G609G*^ mice (hereafter called “progeria mice”) was 23 weeks (Fig. 1A), which was higher when compared to the original description of the mice (14.7 weeks) [26]. This could be at least partly explained by our mice receiving soft food by cage floor feeding at weaning (3 weeks of age) and onwards. Progeria mice also showed a stunted weight gain from 7 weeks of age (Fig. 1B). Reduced lifespan (median survival 41 weeks) and growth were also observed in *Lmna*^*G609G/*+^ mice at a more advanced age (Additional file 2: Fig. S1A-B).

The aortic vascular wall of *Lmna*^*G609G/G609G*^ mice has been shown to undergo extensive VSMC loss [26]. VSMC density quantification in the aortic arch of *Lmna*^*G609G/G609G*^ mice from ages 4 to 15 weeks allowed us to characterize the pattern of VSMC loss. VSMC reduction was progressive, started at 7 weeks of age and became significant in 10 week-old mice, with a 38.6% reduction (Fig. 1C-E). By 15 weeks of age, only 15.6% of the VSMCs remained in the aortic arch of *Lmna*^*G609G/G609G*^ mice (Fig. 1D-E).

We next wondered if the observed VSMC loss was sufficient to trigger a regenerative response from the surviving VSMCs. Using immunostaining, we detected a transient increase in the number of VSMCs positive for the proliferative markers Ki67 and Pcna in progeria mice between 6 and 10 weeks of age. A peak in proliferative marker expression was detected in 8-week-old progeria mice, as 1.85% and 10.27% of VSMCs were positive for Ki67 (Fig. 1F-G) and Pcna (Fig. 1H-I), respectively.

We also evaluated the levels of apoptosis using TUNEL assay. The latter showed a significant increase in apoptosis in 12-weeks old progeria mice, with 4.48% of TUNEL-positive VSMCs (Fig. 1J-K), coinciding with the strongest reduction in VSMC density (Fig. 1C-E).

Overall, the aortic arch of progeria mice was progressively depleted of VSMC coverage, coinciding with a significant increase in apoptosis. A transient surge in VSMCs positive for proliferative markers was also observed. Taken together, these results formed the basis for the timepoints included in the following parts of the study.

### Disease-enriched VSMC and fibroblast populations are present in the aortic arch from progeria mice

**Fig. 2:**
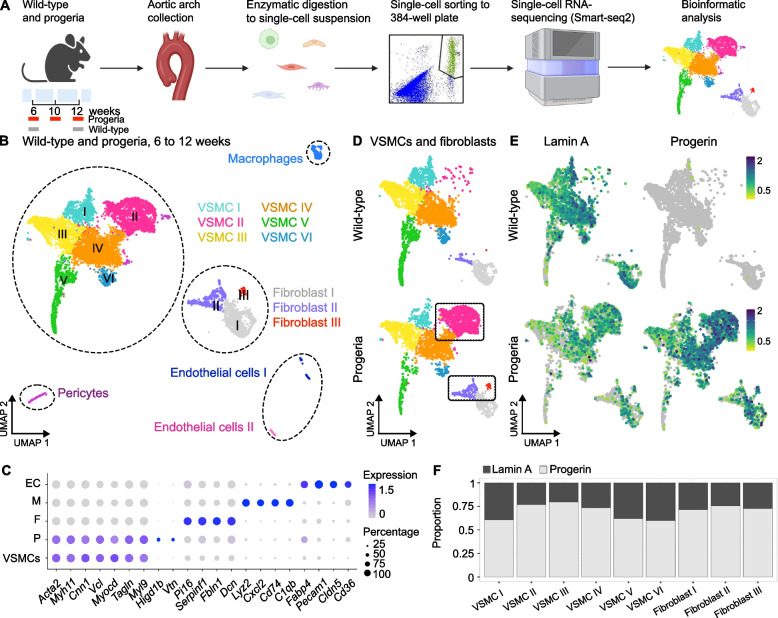
Single-cell RNA-sequencing (Smart-seq2) analysis identifies VSMC and fibroblast subpopulations enriched in progeria mice. **A** Schematic representation of the experimental design. Aortic arches from wild-type and progeria mice were collected at 6, 10 and 12 weeks of age for progeria mice and at 6 and 12 weeks for wild-type mice. A single-cell suspension was generated by enzymatic digestion (VSMCs, fibroblasts, pericytes, endothelial cells and macrophages are illustrated in red, blue, purple, orange and green, respectively). Subsequently, live cells were individually sorted to a 384-well plate for scRNA-seq using Smart-seq2, and bioinformatic analysis was performed. Created in BioRender. Eriksson, M. (2026) https://BioRender.com/h434pj0**B** UMAP visualization of 8968 vascular cells derived from both wild-type and progeria mice of all the collected ages. The five main cell types identified in the aortic arch are indicated. **C** Dotplot showing the expression levels of the genes used to annotate the cell identity assigned in B. Dot size indicates the percentage of cells expressing a particular gene and the intensity of the color indicates the average log-normalized gene expression levels of the given gene. EC = endothelial cells, M = macrophages, F = fibroblasts, P = pericytes, VSMCs = vascular smooth muscle cells. **D** UMAP visualization of the clustering results based on mouse genotype. Only VSMCs and fibroblasts are shown, and all the collected ages are included. The progeria-enriched clusters are highlighted with a dotted box. **E** UMAP visualizations of VSMCs and fibroblasts highlighting log10 of lamin A (left) and progerin (right) transcript pseudocounts (counts + 1) in wild-type (up) and progeria (down) cells. The color scale applies to cells in which at least 1 read was detected. Cells in which no progerin or lamin A specific reads were detected are labelled in grey. Only VSMCs and fibroblasts of all collected ages are shown. **F** Barplot showing the proportion of lamin A and progerin relative to the sum of both isoforms in all VSMC and fibroblasts populations

To understand the molecular mechanisms leading to VSMC loss in the progeria mice, we performed scRNA-seq in the aortic arch of progeria mice at different stages of the VSMC loss progression: age 6 weeks (prior to the start of the VSMC loss), 10 weeks (moderate VSMC loss) and 12 weeks (advanced VSMC loss). The aortic arch from 6 and 12-weeks old wild-type mice was used as control. The aortic arches were enzymatically digested to yield a single-cell suspension of vascular cells, from which live cells were individually sorted into 384-well plates using FACS (Fig. 2A). In addition, some progeria mice further carried the *Acta2*-GFP transgene, allowing an enrichment of VSMCs by performing FACS of GFP-positive cells. Following sequencing and quality control (see Methods), we analyzed 3922 wild-type and 5046 progeria cells. Joint clustering of wild-type and progeria cells using Seurat revealed 5 main cell populations in the murine aortic arch (Fig. 2B). Using known markers of vascular cell identity (Fig. 2C), we classified these 5 populations as macrophages, VSMCs, fibroblasts, endothelial cells (EC) and pericytes (Fig. 2B, C). In addition, we could further subdivide the VSMCs clusters in 6 different subpopulations (VSMC I-VI), the fibroblasts into 3 subpopulations (Fibroblast I-III) and ECs into 2 subpopulations (Endothelial cells I-II; corresponding to periaortic microvascular EC and aortic lumen EC, respectively [65]) (Fig. 2B, Additional file 1: Table S2). Next, we compared the abundance of wild-type and progeria cells in the most abundant cell types, VSMCs and fibroblasts, revealing an enrichment of progeria cells in subpopulations VSMC II, fibroblast II and fibroblast III (Fig. 2D, Additional file 2: Fig. S2A). In particular, 97.5% of VSMC II, 87.2% of fibroblast II and 98.7% of fibroblast III cells were progeria cells. Thus, we show that the vasculature of progeria mice displayed a characteristic transcriptomic profile. We have deposited the gene expression data as a user-friendly searchable database accessible by clicking the following link: Progeria database.

### Detection of the progerin transcript in progeria VSMCs and fibroblasts

Next, we took advantage of the full-length transcript information from Smart-seq2 to assess the levels of the progerin transcript in individual cells. We first evaluated global *Lmna* gene expression levels, and we observed that 98.7% and 99.97% of wild-type and progeria cells, respectively, expressed *Lmna* (Additional file 1: Table S3), indicating robust gene detection across both genotypes.

Subsequently, we performed targeted detection of the progerin and Lamin A transcripts within the sequencing data. For this, we aligned the sequencing reads to reference sequences covering the specific junction of exon 11–12 of either the Lamin A and/or the progerin transcripts (See Methods). Using this approach, we were able to detect the progerin and the lamin A transcripts in 54.1% and 26.4%, respectively, of all progeria cells, compared to 0.21% and 60.4% in wild-type mice (Fig. 2E, Additional file 1: Table S3, Additional file 2: Fig. S2B-C), highlighting the specificity of the strategy. Moreover, we observed that progerin was the predominant isoform compared to Lamin A in all analyzed VSMC and fibroblast subpopulations, although no specific differences in this ratio could be observed between the cell subpopulations (Fig. 2F).

Further analysis confirmed that the reduced detection frequency was due to the strict criteria for read inclusion and not a detection issue. We performed an additional alignment to *Lmna* exons 1 and 11, and the 150 bp from exon 11 deleted in progeria mice. These alignments showed widespread expression of *Lmna* exons 1 and 11 in both wild-type (87.66% and 77.92%, respectively) and progeria mice (95.24% and 78.76%), and reduced representation of the 150 bp deleted in progeria mice (32.5%) compared to wild-type (67.39%) (Additional file 1: Table S3). Since VSMCs are key in the vascular phenotype of progeria mice, we confirmed by immunostaining that 100% of progeria VSMCs expressed the progerin protein (Additional file 2: Fig. S2D-E).

In sum, our dataset allowed the specific detection of the progerin and Lamin A transcripts in the aortic arch.

### The disease-enriched VSMC II subpopulation comprises end-stage VSMCs

**Fig. 3:**
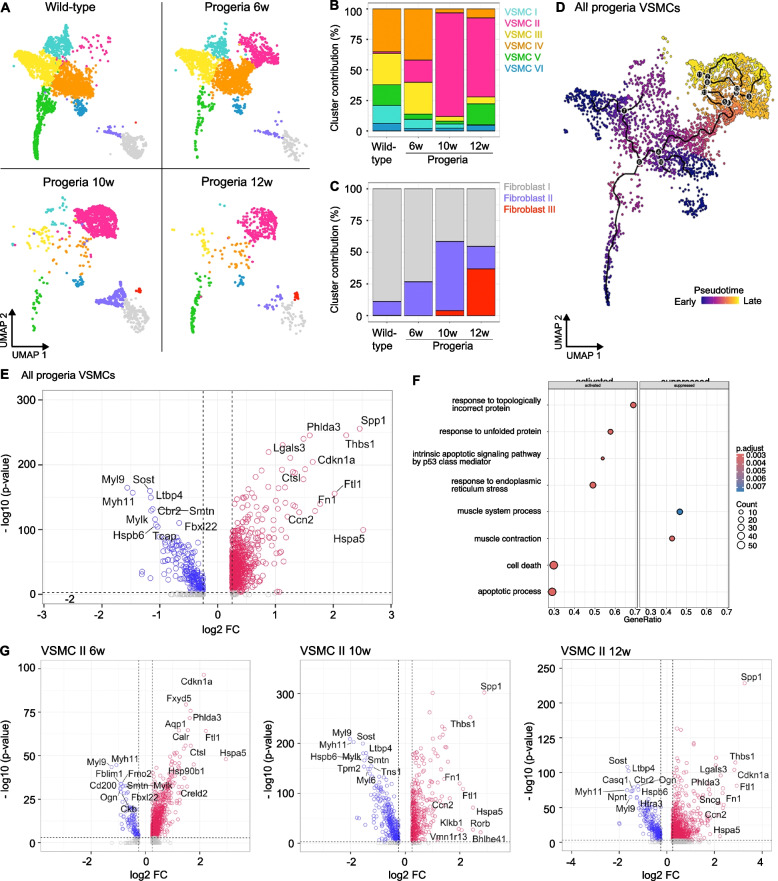
The VSMC and fibroblast populations enriched in progeria mice appear progressively over time. **A** UMAP visualization of the clustering results from wild-type mice and progeria mice of ages 6, 10 and 12 weeks. Only VSMCs and fibroblasts are shown. **B-C** Relative contribution of each VSMC subpopulation to the overall VSMC population (B) and of each fibroblast subpopulation within the fibroblast population in wild-type and progeria mice of ages 6, 10 and 12 weeks (C). **D** Pseudotime and trajectory analysis inferred by applying Monocle3 in VSMCs derived from progeria mice from all collected ages. **E** Volcano plots showing differentially expressed protein coding genes (DEGs) between the VSMC subpopulations shared between wild-type and progeria mice at 6 weeks of age and the VSMC II cells. **F** Selected Gene Ontology terms of interest identified from the DEGs determined in C. The size of the dot represents the gene count, and the color scale (blue-red) the adjusted p-value. **G** Volcano plots showing differentially expressed genes (DEGs) between the VSMC subpopulations shared between wild-type and progeria mice at 6 weeks of age and the VSMC II cells at ages 6 weeks (*left*), 10 weeks (*middle*) and 12 weeks (*right*). For the volcano plots in E and G, the Wilcoxon Rank Sum test was used by the FindMarkers function was used to identify the DEGs between two groups. Dots in red represent genes with log2 FC > 0.25 and -log10 (p-value) > 3. Dots in blue represent genes with log2 FC < −0.25 and -log10 (*p*-value) > 3

Subsequently, we determined if the frequency of the progeria-enriched VSMC and fibroblast populations varied with the age of the progeria mice. Visualization of the clustering of VSMCs and fibroblasts from the progeria mice by age (Fig. 3A) revealed that VSMC II cells were already present at 6 weeks of age, representing 18% of all VSMCs, compared to 1% in the wild-type mice. By the age of 10 weeks, the frequency of VSMC II cells was increased to 84.9%, but it was later reduced to 64.7% in 12-week-old progeria mice. This was paralleled by a progressive reduction in the frequency of most of the remaining VSMC subpopulations (Fig. 3B and Additional file 1: Table S4). Fibroblast II cells were present in 6 weeks old progeria mice (26.7% of all fibroblasts), reaching their maximum frequency at 10 weeks (54.5%). Fibroblasts III were first detected in 10 week old progeria mice at a frequency of 4%, which later reached 36.9% by 12 weeks of age (Fig. 3C and Additional file 1: Table S4). Given that GFP sorting could induce differences in cell-type and subpopulation frequencies (Additional file 1: Table S5), we ensured that these relative frequencies were not biased by GFP sorting by performing a downsampled analysis using a matched number of GFP-sorted and non-sorted cells. We observed similar age-associated subpopulation shifts in the VSMCs and fibroblasts from the GFP-sorted and non-sorted cells upon downsampling, confirming GFP sorting was not influencing the frequency results (Additional file 1: Table S6).

Trajectory analysis using Monocle3 highlighted VSMC II cells as an endpoint for all remaining subpopulations but also showed heterogeneity within VSMC II population (Fig. 3D). To understand the differences between progeria-enriched VSMCs and other VSMCs, we first compared gene expression between VSMC II cells and the remaining progeria VSMC subpopulations shared with the wild-type mice. This analysis showed *Cdkn1a*, *Hspa5*, *Thbs1* or *Spp1* in the top upregulated genes, together with *Myh11*, *Myl9* or *Smtn* in the top downregulated genes in VSMC II cells (Fig. 3E, Additional file 1: Table S7). Gene Ontology (GO) analysis revealed an enrichment in terms related to the proteotoxic response (e.g. “response to unfolded protein”, “response to topologically incorrect protein”), apoptosis (e.g. “cell death”, “apoptotic process”, “intrinsic apoptotic signaling pathway by p53-class mediator”), and loss of muscle function (e.g. “muscle contraction”, “muscle system process”) (Fig. 3F, Additional file 1: Table S8).

To assess if these transcriptional changes were exclusive to VSMC II cells rather than a generalized feature of all progeria VSMCs, even if to a lesser extent, we further compared gene expression between each VSMC subpopulation in the progeria mice and the correspondent subpopulation in wild-type mice at 6 weeks of age. Overall, we found no evidence of a widespread increased proteotoxic response or loss of VSMC identity, although we observed a generalized increase in *Cdkn1a* levels. VSMC V cells were enriched in unknown transcripts and were therefore not analyzed further. In VSMC subpopulations I, III and IV, we observed increased expression of ECM-related and aging genes such as *Sparc* [66] and *Mfge8* [67] (Additional file 1: Table S9, Additional file 2: Fig. S3A-C), which may reflect early vascular dysfunction or an adaptive response to VSMC loss.

In contrast, the VSMC VI subpopulation exhibited virtually no relevant protein coding DEGs (Additional file 1: Table S9). We initially hypothesized that this could be due to a low frequency of VSMC VI cells in which the progerin transcript was detected. However, 70.45% of VSMC VI cells had progerin-specific reads (Additional file 1: Table S10), indicating that the absence of transcriptional alterations likely reflects the intrinsic characteristics of this subpopulation. In sum, we identified a distinct VSMC subpopulation (VSMC II) in the progeria mice with unique transcriptional features.

### Age-dependent transcriptional evolution of the disease-enriched VSMC II subpopulation

Given the differences in the frequency of VSMC II cells with age and the trajectory analysis, we next assessed how the transcriptional profile of VSMC II cells evolved with age. For this purpose, we compared gene expression between the progeria cells in the VSMC II subpopulation at 6, 10 and 12 weeks with the remaining progeria VSMCs (I, III, IV, V and VI), but the latter only at 6 weeks of age, when the cell composition was similar to that of wild-type mice. This was followed by GO term enrichment analysis. We observed that the transcriptional proteotoxic response was present as early as 6 weeks, as shown by the upregulation of genes like *Hspa5, Cdkn1a, Hsp90b1* or *Creld2* (Fig. 3G, Additional file 1: Table S11) and the enrichment in GO terms related to ER stress, the endoplasmic reticulum-associated degradation (ERAD) pathway or an unfolded protein response (Additional file 1: Table S12). Consistent upregulation of ER stress-related genes was observed at the 3 studied ages. A transcriptional profile of VSMC identity loss was also detected from 6 weeks, characterized by a downregulation of contractile genes such as *Myh11* or *Myl9* (Fig. 3G, Additional file 1: Table S11) and the suppression of 1 GO term connected to contraction (“contractile actin filament fiber”) (Additional file 1: Table S12). VSMC dedifferentiation features intensified by 10 weeks of age, with further upregulation of phenotypic switch genes such as *Spp1, Thbs1* or *Fn1* and suppression of 11 GO terms related to muscle function (Fig. 3G, Additional file 1: Table S11, Additional file 1: Table S12). Overall, progeria VSMCs show a progressive transformation into a distinct population with features of ER stress and VSMC dedifferentiation appearing at different ages.

### A subset of VSMC II cells show elevated ER stress and apoptotic signaling

**Fig. 4:**
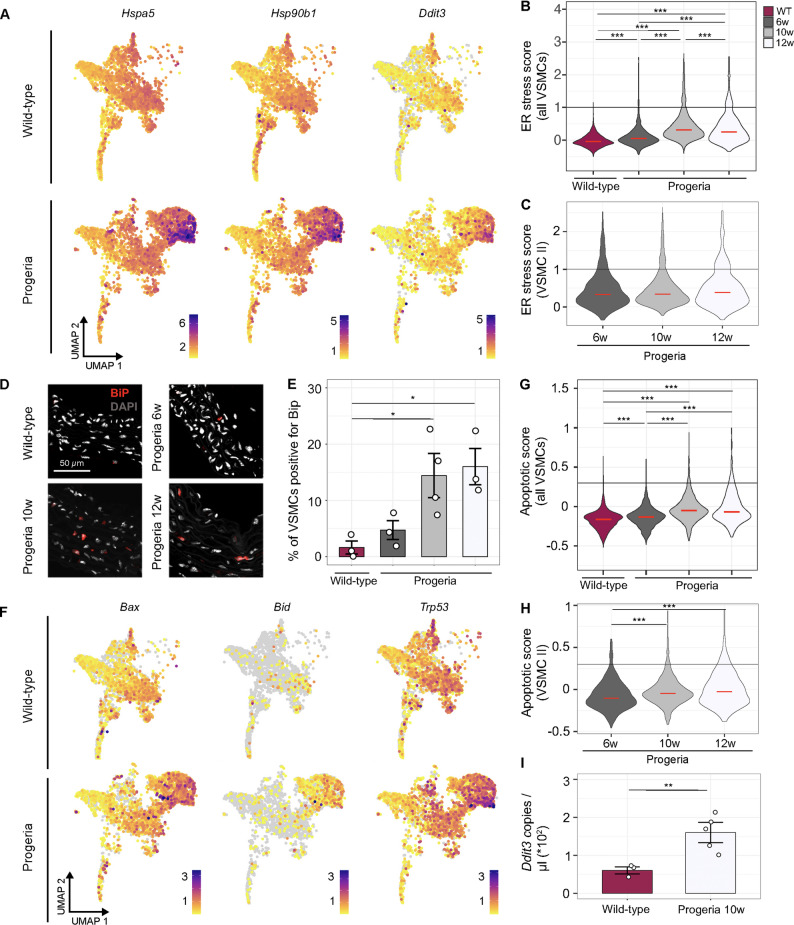
The VSMC II subpopulation shows high levels of ER stress and apoptotic markers. **A.** UMAP plots showing the log-normalized gene expression levels of the ER stress genes *Hspa5*, *Hsp90b1* and *Ddit3* in wild-type and progeria mice of all collected ages. **B.** Violin plots showing the levels of a custom ER stress score (*Hspa5*, *Hsp90b1*, *Atf4*, *Ddit3*, *Sdf2l1*, *Hmox1*, *Gdf15*, *Asns*) in wild-type and progeria VSMCs at ages 6, 10 and 12 weeks. Horizontal line represents a custom-defined threshold to consider a cell ER stress (score > 1). **C.** Violin plots showing the levels of the custom ER stress score from B only in cells from the VSMC II subpopulation. Horizontal line represents a custom-defined threshold to consider a cell ER stress (score > 1). **D.** Immunofluorescence for BiP (*Hspa5*) in aortic arch sections from wild-type (*n* = 3) and progeria mice at ages 6 (*n* = 3), 10 (*n* = 4) and 12 (*n* = 3) weeks. Red = BiP, white = nuclear staining. **E.** Quantification of the BiP staining shown in panel D. **F.** UMAP plots showing the log-normalized gene expression levels of the apoptotic genes *Bax*, *Bid* and *Trp53* in wild-type and progeria mice of all collected ages. **G.** Violin plots showing the levels of a custom apoptosis score (*Bax, Fas, Trp53, Bad, Bid, Pmaip1*) in wild-type and progeria VSMCs at ages 6, 10 and 12 weeks. Horizontal line represents a custom-defined threshold to consider a cell apoptotic (score > 0.3). **H.** Violin plots showing the levels of the custom apoptosis score from G only in cells from the VSMC II subpopulation. Horizontal line represents a custom-defined threshold to consider a cell apoptotic (score > 0.3). **I.** Quantification of *Ddit3* transcript levels (copies/µl) by ddPCR in the aortic arch of wild-type (*n* = 3) and progeria mice of 10 weeks of age (*n* = 5). For panels B, C, G and H, the Kruskal–Wallis test was used. When significant, it was followed by pairwise Wilcoxon rank-sum tests with Bonferroni correction for multiple testing. The red line marks the median. For panel E, one-way ANOVA and Tukey´s Honest Significance tests were used. For panel I, unpaired two-tailed *t*-test with Welch’s correction was used. In panels D and I, mean ± SEM are shown. * = *p*-value < 0.05; ** = *p*-value < 0.01; *** = *p*-value < 0.001. Scale bar in D indicates 50 µm

To assess the extent of the ER stress increase in VSMC II cells, we visualized in the UMAP projection the levels of several ER stress-related genes, such as *Hspa5, Hsp90b1* and *Ddit3* (Fig. 4A). This confirmed that ER stress was largely confined to VSMC II cells, rather than being a widespread feature of all progeria VSMCs. Using a custom ER stress score with highly expressed ER stress genes (*Hspa5*, *Hsp90b1*, *Atf4*, *Ddit3*, *Sdf2l1*, *Hmox1*, *Gdf15* and *Asns*) we observed a progressive increase in

the proportion of all progeria VSMCs with elevated ER stress levels from 6 to 10 weeks, with a mild decrease at 12 weeks (Fig. 4B). Based on the UMAP visualization of the transcriptional score (Additional file 2: Fig. S4A) we defined as ER-stressed those cells that had an ER stress transcriptional score above 1. By these means, only 0.3% of wild-type VSMCs were considered ER stressed. In progeria mice, the number of ER-stressed VSMCs progressively increased from 2.6% of all VSMCs at 6 weeks to 10.19% in 10 weeks, and 10.9% at 12 weeks of age. Furthermore, within the VSMC II subpopulation, very high levels of ER stress were restricted to a small subset of cells (Fig. 4A), and the strength of the transcriptional ER stress response was not upregulated with age (Fig. 4C). In line with this, the percentage of VSMCs within the VSMC II subpopulation remained around 12% at 6 and 10 weeks of age (11.7% and 12.8%, respectively), with only a mild increase at 12 weeks (16.3%).

Immunohistochemical detection of BiP (*Hspa5*) confirmed the increase in ER-stressed VSMCs in progeria mice at 10 and 12 weeks of age (14.4% and 16% of all VSMCs) compared to wild-type (1.6%) (Fig. 4D-E).

Given that the fraction of cells with high ER stress remained stable within the VSMC II subpopulation (Fig. 4C) and that we observed progressive apoptosis in the aortic arch from progeria mice (Fig. 1J-K), we hypothesized that VSMC death in the progeria mice could partly result from elevated ER stress. Visualization of the expression levels of *Bax, Bid* and *Trp53* in the UMAP projection revealed enhanced apoptotic signaling within the VSMC II subpopulation, partially overlapping with high ER stressed cells (Fig. 4F). Accordingly, the proportion of cells with an elevated apoptotic score (*Bax, Fas, Trp53, Bcl2l11, Bad* and *Bid*) increased with age (Fig. 4G). As with the ER stress score, we set as threshold an apoptotic score of 0.3 based on the UMAP for the apoptotic transcriptional score (Additional file 2: Fig. S4B), which defined only 0.21% of wild-type VSMCs as apoptotic. In progeria mice, the number of apoptotic VSMCs increased from 0.92% at 6 weeks of age to 3.67% at 10 weeks and 6.82% by 12 weeks. This increase was also observed within the VSMC II subpopulation: by 6 and 10 weeks of age only 3.95% and 4.12% of VSMC II cells were considered apoptotic, with a steep increase to 8.73% at 12 weeks (Fig. 4H). Finally, ddPCR analysis confirmed increased expression of *Ddit3*, a marker of ER stress-induced apoptosis, in aortas from 10-week old progeria mice (Fig. 4I). Taken together, our data indicate that ER stress in progeria VSMC is not a synchronized, global response, but rather develops at different rates, partially contributing to VSMC death.

### VSMC II cells undergo phenotypic switching towards a fibroblast-like identity

**Fig. 5:**
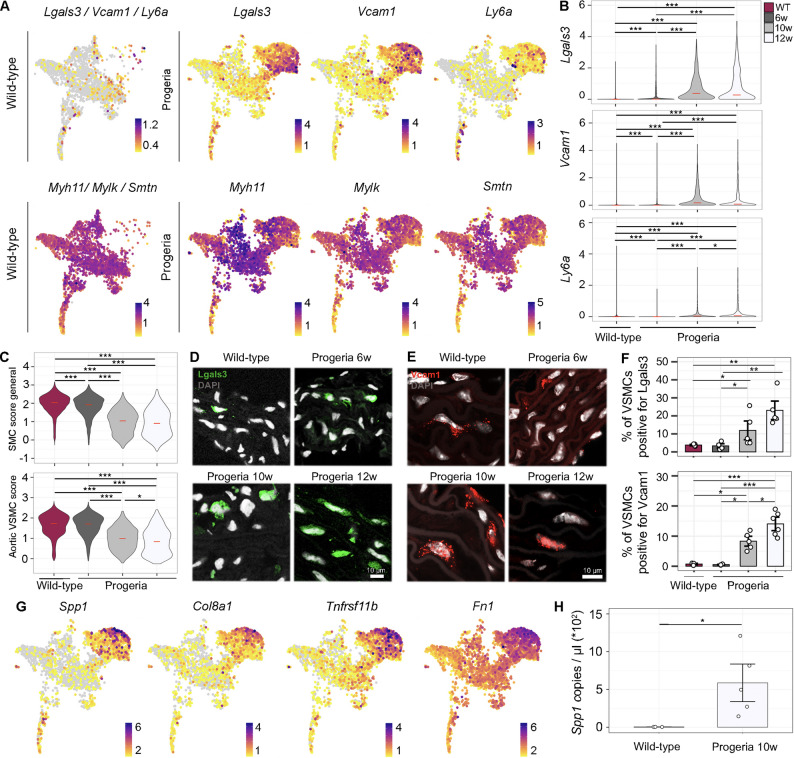
Progeria-enriched VSMC undergo phenotype switching towards a fibroblast-like identity. **A** UMAP plots of VSMCs showing the log-normalized gene expression levels of *Lgals3, Vcam1* and *Ly6a* (dedifferentiation markers, upper plots) as well as *Myh11*, *Mylk* and *Smtn* (contractile markers, lower plots). The expression of each gene is shown individually for the progeria VSMCs and as a combined score for the wild-type VSMCs.** B** Violin plots showing the normalized expression levels of each individual dedifferentiation gene (*Lgals3*, *Vcam1* and *Ly6a*) in wild-type VSMCs as well as VSMC II cells in progeria mice of 6, 10 and 12 weeks of age. **C** Violin plots showing the levels of two scores for contractile identity. *Upper.* A non-tissue specific SMC score (*Acta2, Actg2, Cnn1, Dmpk, Flna, Lmod1, Tagln, Myh11, Mylk, Myl9, Tpm2, Ppp1r12b*). *Lower*. An aortic VSMC identity score (*Tpm1, Mylk, Actn4, Dstn, Sdc4, Itgb1, Smtn, Tagln, Cnn1*) in wild-type VSMCs as well as VSMC II cells in progeria mice of 6, 10 and 12 weeks of age. **D** Immunofluorescence for Lgals3 in aortic arch sections from wild-type (*n* = 3) and progeria mice at ages 6 (*n* = 4), 10 (*n* = 5) and 12 (*n* = 6) weeks. Green = Lgals3, white = nuclear staining. **E** RNAscope for *Vcam1* in aortic arch sections from wild-type (*n* = 3) and progeria mice at ages 6 (*n* = 4), 10 (*n* = 5) and 12 (*n* = 5) weeks. Red = Vcam1 white = nuclear staining. **F** *Upper.* Quantification of the Lgals3 staining shown in panel D. *Lower.* Quantification of the *Vcam1* RNAscope shown in panel E. **G** UMAP plots of VSMCs showing the log-normalized gene expression levels of genes regarding the acquisition of a fibroblast-like identity (*Spp1, Col8a1, Tnfrs11b and Fn1*). **H** Quantification of *Spp1* transcript levels (copies/µl) by ddPCR in the aortic arch of wild-type (*n* = 3) and progeria mice of 10 weeks of age (n = 5). Within panel B, for individual genes, pairwise Wilcoxon rank-sum tests with Bonferroni correction for multiple testing within the FindMarkers function were used. For the contractile score, the data was fetched from the Seurat object and comparisons were manually done using Wilcoxon rank-sum tests with Bonferroni correction for multiple testing. The red line indicates the median. For panel E, Kruskal–Wallis followed by Dunn´s post-hoc test was used, whereas for panel F, one-way ANOVA and Tukey´s Honest Significance tests were used. In panels E, F and H, mean ± SEM are shown. In panel H, unpaired two-tailed t.test was used. * = p-value < 0.05; ** = p-value < 0.01; *** = p-value < 0.001. Scale bar indicates 10 µm

Based on the observation that the *Spp1, Ccl2, Fn1* and *Thbs1* genes were among the top10 upregulated genes in the VSMC II subpopulation (Fig. 3E, Additional file 1: Table S7), together with the enrichment of downregulated GO terms related to muscle differentiation (Fig. 3F, Additional file 1: Table S8), we hypothesized VSMCs were undergoing phenotypic switch. To confirm this, we checked the expression levels of *Lgals3, Vcam1* and *Ly6a*, which have been described as markers of VSMC intermediate states [22], and all 3 genes were significantly upregulated in VSMC II cells (Fig. 5A, Additional file 1: Table S7). The frequency of VSMCs positive for these markers significantly increased by 10 weeks of age (Fig. 5B). Consistent with this transition, VSMC II cell displayed reduced expression of several canonical contractile genes, such as *Myh11*, *Mylk* and *Smtn* (Fig. 5A).

To further quantify the loss of VSMC identity, we calculated two complementary VSMC identity scores: one based on genes characteristic of non-tissue specific SMC identity (*Acta2, Actg2, Cnn1, Dmpk, Flna, Lmod1, Tagln, Myh11, Mylk, Myl9, Tpm2, Ppp1r12b*) [48], and another derived specifically from aortic VSMC genes [23] (*Myh11, Mylk, Smtn, Tpm1, Mylk, Actn, Dstn, Sdc4, Itgb1, Smtn, Tagln, Cnn1*). Both scores progressively decreased from 10 weeks of age (Fig. 5C). We validated these findings by Lgals3 immunostaining (Fig. 5D) and in situ hybridization for *Vcam1* (Fig. 5E). Higher numbers of Lgals3-positive VSMCs were present in the aortic arch of progeria mice at ages 10 and 12 weeks (11.95% and 23.08% of total VSMCs, respectively), compared to wild-type mice (3.8%) (Fig. 5D and 5 F). We also detected a higher frequency of *Vcam1* transcript-positive VSMCs in the progeria mice from 10 and 12 weeks of age (8.32% and 14.06%) than in wild-type mice (0.72%) (Fig. 5E and 5 F).

Furthermore, the increased expression of *Spp1, Col8a1, Tnfrsf11b* and *Fn1* (Fig. 5G) [20–22] suggested that VSMC II cells transition into a fibroblast-like phenotype from 10 weeks of age (Fig. 5G, Additional file 1: Table S11). In agreement with this, aortas from 10-week old progeria mice showed higher *Spp1* expression (Fig. 5H).

Phenotypic switch is key in atherosclerosis, the leading cause of death in HGPS patients for which the underlying susceptibility is largely unknown. To expand on this, we assessed if the effects of progerin on the murine VSMC II population were also active in VSMCs of the arterial wall in patients with coronary artery disease (CAD). For this purpose, we sought enrichments of the differentially expressed VSMC II genes identified in the progeria mice in gene regulatory networks (GRNs) causally linked to CAD in the Stockholm-Tartu Atherosclerosis Reverse Engineering Task (STARNET) study [68]. Interestingly, out of 224 metabolic and arterial wall GRNs in STARNET (starnet.mssm.edu), 4 of the top-6 most VSMC II gene-enriched GRNs (False Discovery Rate (FDR) < 5.7515e-18) were from the arterial wall (Additional file 1: Table S13). Out of these, the atherosclerotic arterial wall GRN39 with 182 genes (whereof 34 from the VSMC II DEGs) stood out by contributing to 1.79% of broad sense CAD heritability and being strongly associated with CAD severity (i.e., SYNTAX score,

P = -log10 × 6). Additionally, as outlined in a more recent STARNET study [69], GRN39 was independently and experimentally validated as a VSMC-specific GRN regulating ECM organization and intracellular calcium levels and suggestively modifying Wnt signaling through the non-canonical Wnt/calcium pathway, which activates SMC proliferation and cell adhesion, promoting atherosclerosis.

Together, these results indicate that the loss of contractile properties and the induction of phenotypic switch in VSMC II cells observed in our progeria mouse model mimic that of VSMCs in humans with CAD, suggesting VSMCs HGPS patients are susceptible to atherosclerotic plaque formation independent of blood lipid levels.

### Computational analysis predicts that phenotypic switching can be triggered through a non-cell autonomous mechanism in progeria

**Fig. 6:**
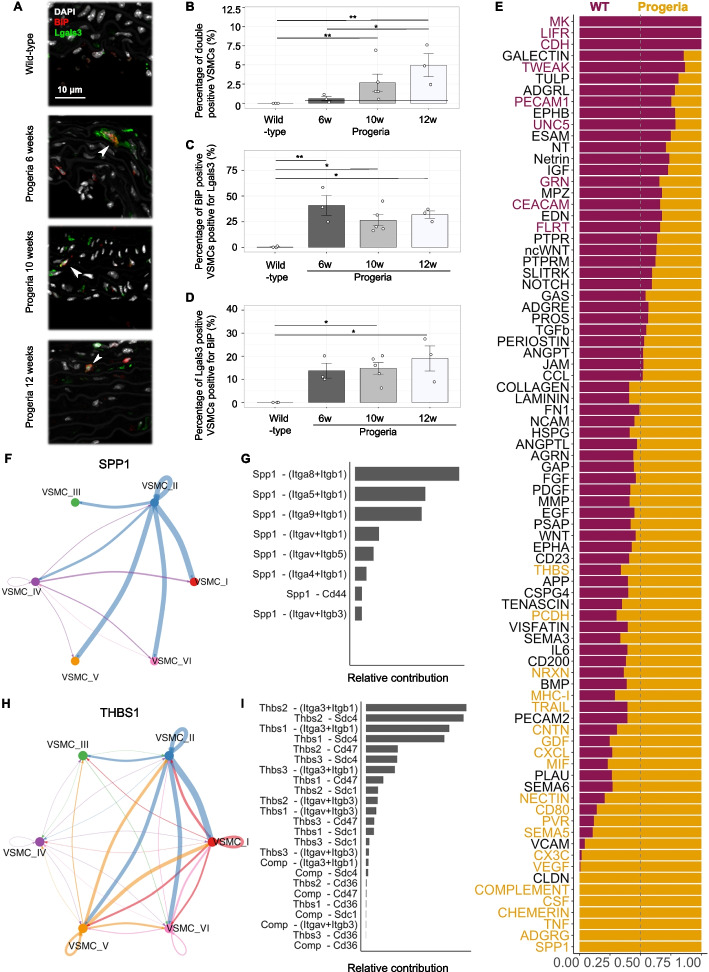
Cell to cell communication modeling predicts that the phenotypic switch can be triggered in a non-cell autonomous mechanism. **A** Immunofluorescence for BiP and Lgals3 in aortic arch sections from wild-type (*n* = 3) and progeria mice at ages 6 (*n* = 3), 10 (*n* = 5) and 12 (n = 3) weeks. Green = Lgals3, red = BiP, white = nuclear staining. Scale bar indicates 10 µm. **B-D** Quantification of the immunofluorescence in A in the form of total number of double positive VSMCs (**B**); BiP-positive VSMCs also positive for Lgals3 (**C**); and Lgals3-positive cells also positive for BiP (**D**).** E** Stacked bar plot showing the information flow of signaling pathways inferred by CellChat in wild-type (pink) and progeria (orange) VSMCs. The color of each segment shows the contribution from each genotype. Pathways highlighted and orange or magenta are significantly different between progeria and wild-type VSMCs, respectively. Statistical significance of differences between WT and progeria VSMCs was assessed using a Wilcoxon rank-sum test. **F** Circle plot showing the interactions among VSMC types for the SPP1 signaling pathway. Nodes represent VSMC subpopulations and edge thickness indicates the strength of inferred interactions. **G** Plots showing the contribution of different ligand-receptor pairs to the SPP1 signaling. **H** Circle plot showing the interactions among VSMC types for the THBS1 signaling pathway. **I** Plots showing the contribution of different ligand-receptor pairs to the THBS1 signaling. In panels B-D, mean ± SEM are shown. For panel B Kruskal–Wallis followed by Dunn´s post-hoc test was used, whereas for panel C and D, one-way ANOVA and Tukey´s Honest Significance tests were used. * = *p*-value < 0.05; ** = *p*-value < 0.01; *** = *p*-value < 0.001

Elevated ER stress due to cholesterol loading levels has been described to lead to Lgals3-positive VSMCs [70]. To assess if this was occurring in our progeria model, we performed immunohistochemistry to identify VSMCs positive for both Lgals3 and BiP. We observed a significant increase in double-positive VSMCs in the 10 and 12-week old progeria mice (2.66% and 4.97% of total VSMCs, respectively) compared to wild-type mice, in which none were detected (Fig. 6A-B). Notably, within BiP-positive VSMCs, Lgals3-positive cells were significantly increased at 6 weeks (40.84% of BiP-positive VSMCs) compared to wild-type mice (0.33%) (Fig. 6A and 6 C). This proportion remained relatively stable with age, consistent with the idea that highly ER-stressed, Lgals3-expressing cells could be prone to death and not accumulate. However, only 13.77% of Lgals3-positive VSMCs were also positive for BiP at 6 weeks of age (Fig. 6A, 6D), and this fraction remained constant with age, suggesting that the phenotypic switch could occur independently of the ER stress.

To test in vivo whether ER stress contributed to the phenotypic switch, we treated progeria mice with tauroursodeoxycholic acid (TUDCA), a chemical chaperone that alleviates ER stress-induced death [71], from ages 6 to 10 weeks. We then assessed cleaved-caspase 3 (CC3) protein levels, VSMC density, BiP levels, and Lgals3 levels (Additional file 2: Fig. S5A-D) in the aortic arch. TUDCA treatment led to a significant decrease in VSMC cell death compared to PBS-treated mice, reflected in reduced CC3-positive VSMCs (fold change = 0.58) (Additional file 2: Fig. S5A, S5E) and increased VSMC density (fold change = 1.34) (Additional file 2: Fig. S5B, S5F). Nevertheless, TUDCA treatment did not result in a significant reduction in BiP-positive VSMCs (Additional file 2: Fig. S5C, S5G), compared to PBS-injected mice. Similarly, the number of Lgals3-positive VSMCs did not show a significant reduction following TUDCA treatment (Additional file 2: Fig. S5D, S5H). Together, these findings indicate that while TUDCA modulates VSMC survival in this model, it does not produce a robust effect on canonical ER stress markers or Lgals3 expression under the conditions tested. Therefore, our data do not support a direct link between VSMC phenotypic switching and ER stress–associated cell death in this model.

We next hypothesized phenotypic switch induction may be partially driven by non-cell autonomous mechanisms. Using CellChat [40] to infer differential communication networks between the VSMCs in wild-type and progeria mice, we found 21 signaling pathways significantly enriched in progeria mice (Fig. 6E). Manual inspection revealed the SPP1, CSF, VEGF, GDF and THBS pathways were predominantly mediated by the VSMC II subpopulation. Since *Spp1* and *Thbs1* expression is characteristic of phenotypic switching, we further used CellChat to inspect the ligand-receptor interactions of these two pathways. We found that most of the interactions in the SPP1 network involved integrins (Fig. 6F-G). Spp1 secretion from fibroblasts has been described to interact with Itga8/Itgb1 and induce phenotype switching [72]. THBS signaling was mostly mediated by Thbs1-3 interacting with different surface proteins (Fig. 6H, I). Thbs1/2 are known to modulate VSMC proliferation and migration, both features of phenotypic switching [73]. Collectively, this computational prediction suggests the phenotypic switch could potentially be triggered in additional cells in a non-autonomous way.

### Progeria VSMCs show a transcriptional ROS response with increased DNA damage

**Fig. 7:**
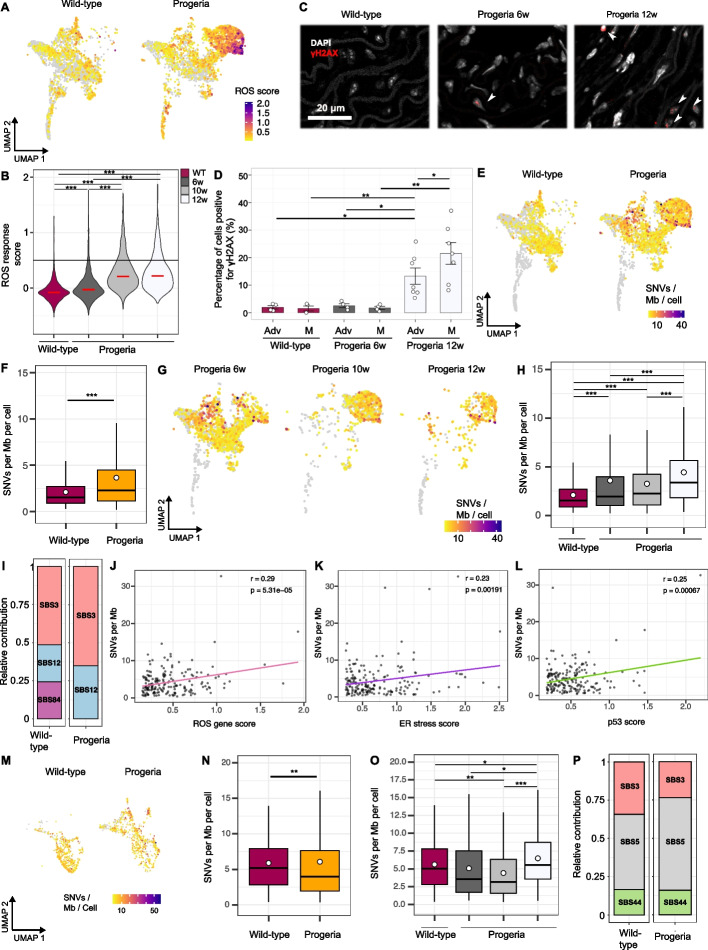
Progeria VSMCs accumulate a higher number of somatic mutations than wild-type VSMCs, correlating with high ER stress, a ROS response and p53 induction, whereas progeria fibroblasts showed a delayed increase in somatic mutations. **A** UMAP plots of wild-type and progeria VSMCs showing the levels of a custom expression score of a ROS transcriptional response score (as *Ftl1*, *Gclm*, *Hmox1*, *Sesn2, Srxn1*, *Txnrd1* or *Txnl1*). **B** Violin plots showing the levels of the custom ROS transcriptional response score in wild-type and progeria VSMCs at ages 6, 10 and 12 weeks. The red line indicates the median. Horizontal line represents a custom-defined threshold to consider a cell ROS-high (score > 0.5). **C** Immunofluorescence for γH2AX in aortic arch sections from wild-type (*n* = 3) and progeria mice at ages 6 (*n* = 4), and 12 (*n* = 7) weeks. Red = γH2AX, blue = nuclear staining. Arrowheads point to examples of positive cells. Scale bar indicates 20 µm. **D** Quantification of the proportion of VSMCs (M, media) and adventitial cells (Adv) positive of γH2AX. Mean ± SEM are shown. Paired t-test was used when comparing media and adventitia from the same age, whereas one-way ANOVA followed by TukeyHSD was applied when comparing the same arterial layer across different mice ages. **E** UMAP plots of wild-type and progeria VSMCs showing the number of mutations per megabase (Mb) in each cell. All collected ages are included. **F** Boxplot reflecting the median and mean (white dot) number of mutations per Mb per cell in each genotype. Only VSMCs with more than 0 mutations were included. **G** UMAP plots of progeria VSMCs showing the number of mutations per megabase (Mb) in each cell, visualized by age. **H** Boxplot reflecting the median and mean (white dot) number of mutations per Mb per cell in each genotype, and by age in the progeria mice. Only VSMCs with more than 0 mutations were included. **I.** Main mutational signatures identified in wild-type and progeria VSMCs and their contribution to the overall mutational spectrum **J–L** Scatterplots showing correlations between the number of mutations per Mb per cell and either a custom ROS response module score (*Asns, Atf4, Ddit3, Gdf15, Hmox1, Hsp90b1, Hspa5, Sdf2l1*) (*J*) or the UPR response score shown in panel A (K) or a p53 induced response defined by the expression of *Trp53, Mdm2, Cdkn1a, Gadd45a, Zmat3,* and *Trp53inp1* (L). Only cells with an ER stress gene score/ROS response gene score/p53 gene score above the median were included. The Pearson correlation coefficient (r) is shown on each plot. **M** UMAP plots of wild-type and progeria fibroblasts showing the number of mutations per megabase (Mb) in each cell. All collected ages are included.** N** Boxplot reflecting the median and mean (white dot) number of mutations per Mb per cell in each genotype. Only fibroblasts with more than 0 mutations were included**. O** Boxplot reflecting the median and mean (white dot) number of mutations per Mb per cell in each genotype, and by age in the progeria mice. Only Fibroblasts with more than 0 mutations were included.** P** Main mutational signatures identified in wild-type and progeria fibroblasts and their contribution to the overall mutational spectrum. For the ROS transcriptional response score, the data was fetched from the Seurat object and comparisons were manually done using Wilcoxon rank-sum tests with Bonferroni correction for multiple testing. * = *p*-value < 0.05; ** = *p*-value < 0.01; *** = *p*-value < 0.001. For comparing the number of mutations per Mb per cell between genotypes, the Wilcoxon rank-sum test was used. When comparing across different ages, Kruskal–Wallis followed by pairwise Wilcoxon rank-sum was used

Previous studies have shown that progerin-expressing fibroblasts accumulate high levels of reactive oxygen species (ROS), leading to pronounced cellular consequences [41, 74, 75]. In addition, ER stress is known to promote ROS accumulation [76]. Therefore, we assessed whether disease-enriched VSMCs exhibited signs of a ROS response. Among the significantly upregulated genes in VSMC II cells, we identified several genes characteristic of an antioxidant response, such as *Ftl1*, *Gclm*, *Hmox1*, *Sesn2, Srxn1*, *Txnrd1* or *Txnl1* (Additional file 1: Table S7). Moreover, using a combined expression score of these genes, we observed that the ROS response score was progressively increased in progeria VSMCs and progressed with age (Fig. 7A-B). By defining a UMAP-based threshold of 0.5 for the ROS transcriptional score, we classified only 0.4% of wild-type VSMC as with a high ROS score. In contrast, in progeria mice at 6 weeks, this number was increased to 4.51% and showed a further increase to 20.21% and 21.05% at 10 and 12 weeks of age, respectively (Fig. 7B).

One known consequence of ROS accumulation in progerin-expressing fibroblasts is the induction of double-strand breaks (DSBs) and subsequently of DNA damage [77]. To assess this in our model, we performed immunofluorescence for the DNA damage marker γH2AX. We observed a significant accumulation of γH2AX-positive progeria VSMCs (media) and adventitial cells at 12 weeks (21.5% and 13.3%, respectively), compared to wild-type (1.53% and 1.95%) and 6-weeks old progeria mice (1.75% and 2.54%) (Fig. 7C-D). Furthermore, the proportion of γH2AX-positive VSMCs at 12 weeks was significantly higher than that of γH2AX-positive adventitial cells.

### Progeria VSMCs accumulate more somatic mutations than wild-type VSMCs

A well-known consequence of increased ROS and DSB accumulation is the induction of mutations [78]. Therefore, we hypothesized that progeria VSMCs could carry more somatic mutations than their wild-type counterpart. To test this, we performed somatic mutation calling using a recently published algorithm, SComatic, specifically designed for somatic mutation detection in scRNA-seq data (see Methods). Of note, somatic mutation detection was limited to expressed genes due to the usage of scRNA-seq, where only the transcriptome is sequenced. The expressed genome is more protected from accumulating mutations because of transcription coupled-repair, but exome protection has been shown to be reduced during aging [79, 80]. Overall, progeria VSMCs exhibited a significantly higher number of SNVs (mean of non-zero progeria VSMCs = 3.65 SNVs per megabase per cell, median = 2.29) than wild-type VSMCs (mean = 2.1, median = 1.52, p-value compared to progeria mice < 2.2 * e-16) (Fig. 7E-F, Additional file 1: Table S14). The number of somatic SNVs was variable across the VSMC subpopulations (Additional file 2: Fig. S6A), with significantly higher numbers in VSMC I, II and III cells (mean = 4.02, 3.78 and 3.88 SNVs per megabase per cell, respectively; median = 2.3, 2.57 and 2.42, respectively) than VSMC IV and VI (mean = 2.05, 2.17; median = 1.4, 1.78). An elevated number of somatic SNVs could already be detected at 6-weeks in the progeria mice (mean = 3.59, median = 1.94, p-value compared to wild-type mice < 2.2 * e-16), which remained similar at 10 weeks (mean = 3.26, median = 2.26, p-value compared to wild-type mice < 2.2 * e-16) and significantly increased by 12 weeks of age (mean = 4.43, median = 3.38, p-value compared to wild-type mice < 2.2 * e-16) (Fig. 7G-H). Among the 10,273 unique SNVs identified in progeria VSMCs, 471 were detected in at least 2 cells from the same experimental batch (Additional file 1: Table S15), compared with only 76 of 3958 SNVs detected in wild-type VSMC (Additional file 1: Table S16). In addition, we determined clone size by quantifying the number of cells carrying each recurrent SNVs. We observed that progeria VSMCs showed a tendency to form larger clones (3 to 5 cells in size, with a rare 19-cell clone) than wild-type VSMCs (Additional file 1: Table S17). These findings could indicate increased clonal expansion in progeria VSMCs. When annotating the 471 recurring SNVs in progeria VSMCs, 60 were classified as either missense or stop gain variants, suggesting a potential impact on protein function (Additional file 1: Table S18).

In wild-type VSMCs, 13 SNV out of 76 recurrent SNVs were predicted to affect protein function (Additional file 1: Table S19). These recurrent SNVs in wild-type and progeria mice were not present across different mice batches, in line with the hypothesis that they could be recurrent mutations and not mutational hotspots.

Using the *MutationalPatterns* R package [46]. (see Methods), we compared the mutational landscape of progeria and wild-type VSMCs. The mutational landscape of wild-type VSMCs was predominantly characterized by the SBS3, SBS12 and SBS84 signatures. In progeria VSMC it was only dominated by SBS3 and SBS12, which increased their relative contributions compared to wild-type (Fig. 7I). SBS3 is associated with defective DSB repair, and although SBS12 has been linked to liver cancer, it has also been reported to be highly similar to SBS26, a signature linked to defective mismatch repair [81].

Moreover, we investigated whether the ER stress and the ROS transcriptional responses were associated with the accumulation of SNVs. In 12-week old progeria mice, we observed a significant positive correlation between the ROS response score described above and SNVs accumulation (R = 0.29) (Fig. 7J) A similar correlation between a defined ER stress score (*Asns, Ddit3, Gdf15, Hmox1, Hspa5, Hsp90b1, Sdf2l1*) and the number of somatic mutations (R = 0.23), although this correlation was restricted to VSMCs in which the levels of ER stress were above the median of the ER stress score (Fig. 7K), suggesting a link between ER stress, ROS and somatic SNVs accumulation in progeria VSMCs. Lastly, we further observed this type of correlation between the number of somatic SNVs and a p53 transcriptional response (R = 0.25), based on *Trp53, Mdm2, Cdkn1a, Gadd45a, Zmat3,* and *Trp53inp1* expression, indicating a potential connection between the somatic SNVs and senescence, a feature previously described in progeria [82] (Fig. 7L). These correlations were absent in cells with low ER stress levels, a low ROS response or reduced p53 transcriptional activation (Additional file 2: Fig. S6B-D), and when all VSMCs from 12-week old progeria VSMCs were pooled together, with the exception of the p53 correlation, which remained significant but reduced its strength (Additional file 2: Fig. S6E-G).

Finally, we sought human significance for molecular alterations in progeria VSMCs with a high number of somatic mutations by examining enrichments of DEGs from these highly mutated cells (Additional file 1: Table S20; see Methods) in human GRNs from the STARNET study [68]using their browser (starnet-mssm.edu). Notably, among the top-6 most enriched GRNs (Additional file 1: Table S21), we identified the macrophage-specific GRN122 shown to be causally linked to CAD [69]. Since progeria VSMC are undergoing phenotypic switching, the emergence of this macrophage-specific GRN could indicate a connection between the levels of VSMC mutations and susceptibility to a vascular phenotype (i.e., CAD/atherosclerosis) in humans.

### Progeria-enriched fibroblasts show features of activation and chondrocyte activity, with a delayed accumulation of somatic mutations

To characterize the fibroblast subpopulations (Fibroblast II-III), we performed differential gene expression analysis, comparing each disease-enriched fibroblast population to the fibroblast cells shared with the wild-type (Fibroblast I). This analysis revealed Fibroblasts II cells upregulated genes such as *Chad* (Additional file 2: Fig. S7A) and *Acan* (Additional file 1: Table S22), suggesting a chondrocytic behavior. In line with this, Fibroblast II cells upregulated the expression of cartilage-forming collagen isoforms (*Col2a1*, *Col9a1, Col9a2, Col9a3*, *Col11a1*, *Col11a2*) [56] and reduced the levels of additional collagen isoforms (*Col1a1, Col1a2, Col3a1, Col4a1, Col4a2, Col5a1, Col5a2, Col6a1, Col6a2, Col6a3, Col14a1, Col16a1*) (Additional file 2: Fig. S7B). Assessing the expression of the collagen isoforms by age revealed we could only detect the chondrogenic activity in 10 week-old mice (Additional file 2: Fig. S7C-D). Using a custom cartilage transcriptional score with a threshold of 0, only 0.37% of wild-type fibroblasts were classified as high in cartilage levels, as opposed to 2.29%, 33.49% and 8.51% of progeria mice at 6, 10 and 12 weeks, respectively.

In addition, these fibroblasts upregulated *Spp1* expression (Additional file 1: Table S22), indicating a potential contribution to the VSMC phenotypic switch through extracellular signaling, as within VSMCs. As for Fibroblast III cells, we observed that they represented activated fibroblasts, characterized by high expression of activation genes including *Cthrc1*, *Postn or Tnn* [57–59] (Additional file 1: Table S22, Additional file 2: Fig. S7E-F). A transcriptional score defined by *Cthrc1*, *Postn*, *Thbs4*, *Tnc* and *Tnn* showed mild overall fibroblast activation at 6 weeks of age, with a progressively stronger response by the ages of 10 and 12 weeks (Additional file 2: Fig. S7G). Using an activation score threshold of 1, activated fibroblasts were detected exclusively in progeria mice, representing 0.63% and 21.99% of fibroblasts at 10 and 12 weeks of age, respectively. Overall, no features of ER stress, DNA damage, a response to ROS or apoptosis were evident in neither Fibroblast II and III cells (Additional file 1: Table S22).

Notably, progeria fibroblasts showed an overall reduction in somatic SNV accumulation (mean = 6.06 SNVs per megabase, median = 3.98) and compared to wild-type fibroblasts (mean = 5.92, median = 5.17, p-value vs progeria = 0.0026). The SNV load only increased at age 12 weeks (mean = 7.66, median = 5.83, p-value to wild-type = 0.036) (Fig. 7M-O) (Additional file 1: Table S23). This goes in line with the progressive increase in γH2AX with age (Fig. 7D). In addition, Fibroblast I cells showed a significant upregulation of *Cdkn1a* (Additional file 1: Table S22).

Among the 5392 SNVs detected in progeria fibroblasts, 165 mutations were detected in at least 2 cells from the same experimental batch (Additional file 1: Table S24), similarly to 173 recurrent SNVs out of 4164 total detected SNVs in wild-type fibroblasts (Additional file 1: Table S25). 24 recurrent SNVs affecting protein function were detected in progeria fibroblasts (Additional file 1: Table S26), compared to 41 SNVs in their wild-type counterpart (Additional file 1: Table S27). Clone sizes were similar between wild-type and progeria mice (Additional file 1: Table S17). Lastly, the mutational landscape was dominated by the clock-like signature SBS5 [83] (Fig. 7P) in both wild-type and progeria fibroblasts, with higher levels in the latter. Altogether, progeria fibroblasts showed a cartilage-producing and inflammatory phenotype, and delayed and less significant somatic mutation accumulation, highlighting a cell-type specific genomic vulnerability to progerin.

## Discussion

Cardiovascular disease is the main cause of death in HGPS, but the molecular mechanisms driving it are still not fully understood. In this study, we have performed a detailed cellular and molecular characterization of the evolution of the vascular wall in the aortic arch of progeria mice with disease progression. While loss of aortic arch VSMCs has been observed before in progeria mice, our analysis provides an age-specific VSMC density quantification during disease development, showing that VSMC drop-out in our progeria mice occurs progressively. The overall arterial remodeling is characterized by a transient increase in VSMCs expressing Ki67 and Pcna, and an eventual apoptotic increase. 8-week old progeria mice had a higher frequency of VSMCs expressing proliferative markers, which did not seem sufficient to counteract VSMC death long term. In addition, replication fork collapse and replicative stress [84], as well as mitotic catastrophe [15] have been shown to result in progerin-expressing VSMCs death, which could explain why the burst in proliferative marker expression did not lead to sustained VSMC density. Moreover, these proliferative cells could represent dedifferentiated VSMCs, causing a reduction in functional contractile VSMCs. Lastly, we can’t exclude that a subset of VSMCs positive for proliferation-associated markers may instead reflect cells undergoing DNA damage responses rather than successful proliferation.

Our scRNA-seq of the aortic arch of the progeria mice revealed disease-enriched VSMC and fibroblast subpopulations. Progeria-enriched VSMCs (VSMC II), characterized by elevated ER stress and features of dedifferentiation, were already detectable in 6 weeks old mice, preceding significant VSMC loss. In addition, VSMC I-III cells showed altered ECM gene expression and aging features. These observations highlight that VSMC dysfunction in progeria can occur prior to a widespread loss of VSMC density, in line with a previous report of early arterial stiffening in young *Lmna*^*G609G/*+^ mice [85]. Trajectory analysis and high apoptotic gene expression indicated that most VSMCs eventually transition into the VSMC II state and undergo cell death.

While increased ER stress in progeria has been previously described [86], our analysis revealed that elevated ER stress is not widespread through the vascular wall, as we could not detect it in the progeria fibroblasts. This is in agreement with a previous study that showed that progeria endothelial cells do not have upregulated ER stress [87]. Furthermore, we did not observe a homogenous development of ER stress within the VSMCs. This indicates differences in the susceptibility of VSMCs to progerin, potentially dependent on progerin protein accumulation rates, which may vary both across and within VSMC subpopulations, and with cellular age. In addition, it is possible that the strength of this phenotype is lower than previously reported given that our progeria mice are not exposed to an atheroprone environment.

Supporting cellular differences in progerin susceptibility, VSMC I-IV cells decreased in frequency in 12-week old progeria mice but VSMC VI cells did not. Moreover, they did not exhibit pronounced transcriptional changes. VSMC VI cells were characterized by high *Rgs5* levels, a gene that has been described as potential regulator of the VSMC contractile state [88]. This suggests that an *Rgs5*-mediated contractile phenotype could underlie VSMC sensitivity to progerin.

In addition to the elevated ER stress levels, VSMC II cells were undergoing phenotypic switch. At least two different phenotypes were detected: dedifferentiated/synthetic VSMCs, expressing *Lgals3*, *Vcam1* and *Ly6a*; and fibroblast-like VSMCs, expressing *Spp1*, *Col8a1*, *Fn1* and *Tnfrsb11*. Osteogenic VSMCs have also been detected in the arteries of older progeria mice [89]. We did not detect osteogenic markers in our disease-enriched VSMCs, but GRN analysis revealed an enrichment in GRN39, which has been described to be critical for osteogenic transition in atherosclerosis during CAD [69]. Hence, these features could appear later in the disease progression. The presence of phenotypically switched VSMCs, with a similar profile as arterial VSMCs in CAD, supports that the vascular pathology in HGPS extends beyond VSMC loss. Synthetic and fibroblast-like VSMCs secrete inflammatory molecules and remodel the ECM, potentially increasing atherosclerosis susceptibility without hyperlipidemia [90]. Altered ECM composition, including elastin and collagen changes, may further alter arterial stiffness, exacerbating cardiovascular risk. This effect could be reinforced by the disease-enriched fibroblasts, which increased their production of cartilage and inflammatory molecules. Hence therapies aimed solely at restoring VSMC density may be insufficient, as retention of non-contractile VSMCs can also be detrimental.

Since ER stress has been described to increase the frequency of Lgals3-positive VSMCs [70, 91], we investigated whether these processes were mechanistically linked in our model. Although BiP and Lgals3 staining revealed a significant increase in double-positive VSMCs in progeria mice, single-positive VSMCs remained more abundant. Treatment with TUDCA, a compound known to reduce ER-stress related cell death, only had a mild non-significant effect on Lgals3 levels and the ER stress marker BiP, so it was not possible, under these conditions, to link phenotypic switching with ER stress induced death. Additional mechanisms can be involved, like non-cell autonomous induction of the phenotypic switch. Cell–cell communication computational analysis predicted interactions between VSMC II cells themselves and other VSMCs, through Spp1 and Thbs1, both known to affect VSMC proliferation, migration, and phenotypic modulation [72, 73], although more work is required to validate this potential interaction.

Alternatively, phenotypic switching may be protective response to progerin toxicity. For example, phenotypically switched VSMCs in aneurysm display increased autophagy, and loss of autophagy exacerbated ER stress [92]. Moreover, as mentioned before, mechanical changes in the aortic arch could also modulate VSMC phenotype switching [93].

In addition, using SComatic [43] for somatic mutation calling, we detected a higher number of somatic SNVs in progeria VSMCs compared to wild-type VSMCs, a difference that became more pronounced as the mice aged. We hypothesize that the observed increase in somatic SNVs may be related to elevated ER stress levels and oxidative stress, as ROS can induce DNA damage [94]. Of note, ROS levels were not directly measured in this study, so it is not possible to distinguish whether the observed ROS transcriptional score reflects ROS accumulation or ROS buffering and resolution. Nevertheless, ROS accumulation is known to occur in HGPS [74, 75, 77], and VSMCs with a high ROS transcriptional score also exhibited high expression of ER stress-related genes, supporting our hypothesis. Furthermore, progeria VSMCs also showed a higher number of DSBs, which not only have been described to be a consequence of ROS accumulation in progerin-expressing cells, but also they have been reported to activate the usage of the error-prone non-homologous end-joining (NHEJ) pathway [15], a potential source of mutations. In line with this, in highly stressed VSMCs from 12-week old progeria mice, the number of somatic mutations per cell correlated with the expression of ER stress and ROS genes. Moreover, the combined expression of p53 related genes correlated with the accumulation of somatic SNVs in 12-week old progeria VSMCs indicating these mutations could influence cell death or senescence. Consistently, p53-induced senescence has been previously described in progeria fibroblasts [82].

The presence of recurrent SNVs in progeria VSMCs is consistent with the detected proliferation peak, which could indicate replication-induced mutations. This is further reflected in the detected mutational signatures in progeria VSMCs. SBS3 is associated with homologous recombination deficiency and a consequent shift towards NHEJ [83]. SBS12, although of unknown etiology and previously reported in liver samples [95], has been also proposed to correspond to SBS26 due to their high similarity [81]. SBS26 in turn represents defects in mismatch repair, mostly active during DNA replication [83, 95].

By contrast, we did not detect a significant increase in the number of somatic SNVs in the progeria fibroblasts, until 12-weeks of age, in line with these cells exhibiting DSBs accumulation at this age. A potential explanation is that fibroblasts are more proliferative cells, which would allow negative selection to eliminate cells with a high mutation load, a feature described *in vitro* [96]. The reduced number of recurrent SNVs would support a polyclonal fibroblast population. Moreover, the median mutation load of wild-type fibroblasts (5.92 SNVs per megabase per cell) was higher than that of wild-type VSMCs (2.1 SNVs per megabase per cell). This could be explained by the distinct embryonic origins of aortic arch VSMCs (neural crest derived) [97] and fibroblasts (mesoderm derived) [98], but differences in their proliferative rates or environmental exposure should not be excluded. In addition, disease-enriched fibroblasts seemed less susceptible to progerin, consistent with the absence of ER stress, DNA damage, a ROS response and apoptosis features in these cells. Thus, the fibroblast phenotype observed in progeria mice may represent a secondary response to changes occurring within the vascular wall rather than a direct consequence of progerin toxicity. This is in agreement with previous studies that identified fibrosis and an increase in osteogenic markers in mice with VSMC-restricted progerin expression [99, 100], and dermal fibrosis in mice with basal and suprabasal keratinocyte-restricted progerin expression [101].

Overall, we show that somatic SNVs accumulate in a cell type-specific manner in the progeria mice, suggesting that somatic mutations could be connected to HGPS-disease development in the aortic arch. This goes in line with somatic mutation accumulation being a feature of aging. Moreover, we detected recurrent SNVs in VSMCs affecting protein function, suggesting a potential functional effect. Nevertheless, the number of somatic SNVs shown in this study is likely underestimated, as by doing scRNA-seq we are only sequencing the expressed exome, where exons are known to be more protected from mutation accumulation [102].

This limitation in genomic coverage, together with the sparse nature of scRNA-seq, likely attenuates the correlations showcased in Fig. 7J-L. Further studies with higher resolution sequencing techniques for mutation calling are needed to explore the full mutational landscape induced by progerin, as well as the cellular mechanisms underlying somatic mutation accumulation in this context.

Of note, some limitations are present in our study. The distribution of the sex of the mice was not fully balanced across all experimental cohorts, and sex-dependent differences in vascular biology have previously been described [103, 104]. For example, females have more stable fibrous caps, with a stronger VSMC contractile phenotype Nevertheless, similar transcriptional features were observed across independent cohorts with differing sex composition, suggesting our main conclusions regarding VSMC identity and phenotypic switching are unlikely to be primarily driven by sex. However, it is not possible to exclude sex-specific effects on vascular somatic mutagenesis, which remains poorly characterized.

## Conclusions

Altogether, we provide an atlas of the cellular composition and molecular changes along the progression of vascular dysfunction in the progeria mice. The data are publicly available as a user-friendly searchable database, providing a resource for the research community that can not only assist in preclinical trials of HGPS but also facilitate the discovery of novel mechanisms to be targeted to ameliorate cardiovascular disease in HGPS. Our findings on somatic mutation accumulation highlight the need for early HGPS interventions to prevent irreversible tissue damage. Furthermore, cell-type specific responses to progerin indicate these therapies can be targeted to the primarily affected cell-types, like VSMCs. Lastly, since premature aging in HGPS resembles the vascular phenotype of diseases like chronic kidney disease [100], or CAD [68, 69], our data may help deepen the understanding of vascular aging beyond HGPS.

## Supplementary Information


Supplementary Material 1.
Supplementary Material 2.


## Data Availability

The data are deposited in GEO with accession number GSE317471^63^ and can be access through the following link: https://www.ncbi.nlm.nih.gov/geo/query/acc.cgi?acc=GSE317471.
