# Supplementary material for: Single-cell analysis of the progeria arterial wall reveals progerin-induced progressive, cell type-specific dysfunction and somatic mutation accumulation
Source: Genome Med. 2026 Jul 31;18:115. doi: 10.1186/s13073-026-01719-6 (PMC13425876; doi:10.1186/s13073-026-01719-6)
Supplement: Supplementary file 2 — Supplementary Material 2. [file 13073_2026_1719_MOESM2_ESM.docx]

**ADDITIONAL FILE 2**

**Single-cell analysis of the progeria arterial wall reveals progerin-induced progressive, cell type-specific dysfunction and somatic mutation accumulation**

Lara G. Merino^1^, Gwladys Revêchon^1*^, Santhilal Subhash^1,2*^ , Fabiana Stefani^1*^, Daniel Whisenant^1^, Marianna Skipitari^1^, Quentin Giraud^1^, Lars Muhl^1,3^, Giuseppe Mocci^1^, Johan Björkegren^1^, Piotr Machtel^1^, Liqun He^1^, Christer Betsholtz^1^, Maria Eriksson^1^

^1^ Department of Medicine, Huddinge, Karolinska Institutet, Huddinge, Sweden

^2^ Department of Biosciences and Bioengineering, Indian Institute of Technology Jammu, Jammu, India

^3^ Department of Clinical Medicine, Centre for Cancer Biomarkers (CCBIO), University of Bergen, Bergen, Norway.

*These authors contributed equally

Corresponding authors: Maria Eriksson, [maria.eriksson.2@ki.se](mailto:maria.eriksson.2@ki.se) and Lara G. Merino, [lara.garcia.merino@ki.se](mailto:lara.garcia.merino@ki.se)

Postal address corresponding authors:

Karolinska Institutet
Department of Medicine, Huddinge (MedH)
Maria Eriksson and/ Lara G. Merino, Integrative Cardiovascular, Cancer and Ageing Research unit (ICCA)

SE-141 83 Huddinge, Sweden

**Fig. S1**

**Fig. S1. Phenotypic characterization of the *Lmna^G609G^*^/+^ mice.**

**A**. Survival analysis of wild-type (from Osorio et al 2009, Sci Transl Med) and *Lmna^G609G^*^/+^ mice (n=36). Dotted line indicates 50% survival. **B**. Body weight measurements (grams) of wild-type (n=20) and *Lmna^G609G^*^/+^ mice (n=24) for 25 weeks.

**Fig. S2**

**Fig. S2. Differential vascular populations and Lamin A / progerin levels in the aortic arch of progeria mice.**

**A.** UMAP visualization of the clustering results based on the mouse genotype (*left*: wild-type; *right*: progeria mice), showing all identified cell types with their real distance. All collected ages are included. **B** and **C** UMAP visualizations of all progeria cells highlighting log10 of lamin A (B) and progerin (C) transcript pseudocounts (counts + 1) in wild-type (left) and progeria (right) cells. The color scale applies to cells in which at least 1 read was detected. Cells in which no progerin or lamin A specific reads were detected are labelled in grey. Cells from all collected ages are shown. **D.** Immunofluorescence for progerin and smooth-muscle actin in aortic arch sections from wild-type (n = 3) and progeria mice at age 6 weeks (n = 3). Red = Progerin, white = smooth-muscle actin, blue = nuclear staining. **E.** Quantification of the progerin staining shown in panel D. For panel E, the Wilcoxon test was used, mean ± SEM are shown. * = p-value < 0.05. Scale bar in E indicates 20 µm

**Fig. S3**

**Fig. S3. VSMC subpopulations shared between the progeria and wild-type mice do share features of disease-enriched VSMCs. A-C**. Volcano plots showing differentially expressed protein coding genes (DEGs) between the VSMC subpopulations I (A), III (B) and IV (C) in the progeria mice and their wild-type counterpart. The analysis was performed in cells from 6-week old mice. DEGs between progeria and wild-type subpopulations VSMC V and VI were scarce.

The Wilcoxon Rank Sum test was used by the FindMarkers function was used to identify the DEGs between two groups. Dots in red represent genes with log2 FC > 0.25 and -log10 (p-value) > 3. Dots in blue represent genes with log2 FC < 0.25 and -log10 (p-value) > 3.

**Fig. S4**

**Fig. S4.** **The expression of ER stress and apoptotic genes can be summarized in a transcriptional score**.

**A-B**. UMAP plots of wild-type and progeria VSMCs showing the levels of a custom expression score for ER stress (A) and apoptosis (B).

**Fig. S5**

**Fig. S5. TUDCA treatment influences the levels of phenotypic switch.**

**A.** Immunofluorescence of cleaved-caspase 3 (CC3, red) in the aortic arch from wild-type and progeria mice aged 10 weeks that were injected with either PBS or TUDCA 400 mg/kg/day 3 times a week for 4 consecutive weeks. White = nuclear staining. **B.** Nuclear staining (white) in the aortic arch from wild-type and progeria mice injected with either PBS or TUDCA. **C.** Immunofluorescence of BiP (red) in the aortic arch from wild-type and progeria mice injected with either PBS or TUDCA. **D**. Immunofluorescence of Lgals3 (green) in the aortic arch from wild-type and progeria mice injected with PBS or TUDCA. **E-H**. Quantification of CC3 (**E**), VSMC density (**F**), BiP (**G**), and Lgals3-positive VSMCs (**H**) Data are shown as mean ± SEM.

For panels **E-F**, one-way ANOVA followed by followed by Holm–Šidák multiple comparisons test was performed. For panels **G-H**, one-way ANOVA followed by TukeyHSD test was performed.

* = p-value < 0.05; ** = p-value < 0.01; *** = p-value < 0.001. Scale bars in A to F show 50 µm.

**Fig. S6**

**Fig. S6. The number of somatic mutations per cell is variable across the VSMC subpopulation and does correlation with ER stress, ROS response and a p53 transcriptional response in lowly stressed VSMCs**.

**A.** Boxplot reflecting the median and mean (white dot) number of mutations per Mb per cell in each VSMC subpopulation. VSMC V cells are excluded due to their artifactual characteristics. Only VSMCs with more than 0 mutations were included**.** **B-C**. Scatterplots showing correlations between the number of mutations per Mb per cell and either a custom UPR response module score (*Asns, Atf4, Ddit3, Gdf15, Hmox1, Hsp90b1, Hspa5, Sdf2l1*) (**A**); the ROS response score shown in Figure 7A (**B**); or a p53 induced response defined by the expression of *Trp53, Mdm2, Cdkn1a, Gadd45a, Zmat3,* and *Trp53inp1* (**C**). The Pearson correlation coefficient (r) is shown on each plot. Only cells with an ER stress gene score / ROS response gene score / p53 gene score below the median were included. **E-G**. Scatterplots showing correlations between the number of mutations per Mb per cell and either a custom UPR response module score (**E**); the ROS response score shown in Figure 7A (**F**); or a p53 induced response (**G**). The Pearson correlation coefficient (r) is shown on each plot. All cells at age 12 and with SNV load > 0 were included. For comparing the number of mutations per Mb per cell between clusters (**A**), Kruskal-Wallis followed by pairwise Wilcoxon rank-sum with Benjamini-Hochberg correction was used. * = p-value < 0.05; ** = p-value < 0.01; *** = p-value < 0.001.

**Fig. S7**

**Fig. S7. Progeria fibroblasts have a distinct transcriptional profile.**

**A.** Volcano plots showing differentially expressed protein coding genes (DEGs) between the Fibroblast II and Fibroblast I subpopulations. **B.** UMAPs showing in fibroblasts from wild-type and progeria mice of all collected ages the expression levels of a custom score for either chondrocyte-associated collagens (*Col1a1, Col1a2, Col3a1, Col4a1, Col4a2, Col5a1, Col5a2, Col6a1, Col6a2, Col6a3, Col14a1, Col16a1)* or regular fibroblasts collagens (*Col2a1*, *Col9a1, Col9a2, Col9a3*, *Col11a1*, *Col11a2*). **C-D.** Violin plots showing the levels of chondrocyte/normal fibroblast score in wild-type and progeria mice of 6, 10 and 12 weeks of age. Horizontal line indicates a threshold of 0 for the score. **E.** Volcano plots showing differentially expressed protein coding genes (DEGs) between the Fibroblast III and Fibroblast I subpopulations. **F.** UMAPs showing in fibroblasts from wild-type and progeria mice of all collected ages the expression levels of a custom score for fibroblasts activation (*Cthrc1*, *Postn*, *Thbs4*, *Tnc* or *Tnn*). **G.** Violin plots showing the levels of activated fibroblast score in wild-type and progeria mice of 6, 10 and 12 weeks of age. Horizontal line indicates a threshold of 1 for the score

For violin plots, red line represents median. For the volcano plots in A and E, the Wilcoxon Rank Sum test within the FindMarkers function was used to identify the DEGs between two groups. Dots in red represent genes with log2 FC > 0 and -log10 (p-value) > 3. Dots in blue represent genes with log2 FC < 0 and -log10 (p-value) > 3
